# Chromosome-scale genome assembly and annotation of the tetraploid potato cultivar Diacol Capiro adapted to the Andean region

**DOI:** 10.1093/g3journal/jkae139

**Published:** 2024-07-26

**Authors:** Paula H Reyes-Herrera, Diego A Delgadillo-Duran, Mirella Flores-Gonzalez, Lukas A Mueller, Marco A Cristancho, Luz Stella Barrero

**Affiliations:** Corporación Colombiana de Investigación Agropecuaria (AGROSAVIA), Bogotá, Cundinamarca 250047, Colombia; Corporación Colombiana de Investigación Agropecuaria (AGROSAVIA), Bogotá, Cundinamarca 250047, Colombia; Boyce Thompson Institute, Ithaca, NY 14850, USA; Boyce Thompson Institute, Ithaca, NY 14850, USA; Vicerrectoría de Investigación y Creación, Universidad de los Andes, Bogotá 111711, Colombia; Corporación Colombiana de Investigación Agropecuaria (AGROSAVIA), Bogotá, Cundinamarca 250047, Colombia

**Keywords:** genome assembly, *Solanum tuberosum*, Andigenum group, tetraploid assembly

## Abstract

Potato (*Solanum tuberosum*) is an essential crop for food security and is ranked as the third most important crop worldwide for human consumption. The Diacol Capiro cultivar holds the dominant position in Colombian cultivation, primarily catering to the food processing industry. This highly heterozygous, autotetraploid cultivar belongs to the Andigenum group and it stands out for its adaptation to a wide variety of environments spanning altitudes from 1,800 to 3,200 meters above sea level. Here, a chromosome-scale assembly, referred to as DC, is presented for this cultivar. The assembly was generated by combining circular consensus sequencing with proximity ligation Hi-C for the scaffolding and represents 2.369 Gb with 48 pseudochromosomes covering 2,091 Gb and an anchor rate of 88.26%. The reference genome metrics, including an N50 of 50.5 Mb, a BUSCO (Benchmarking Universal Single-Copy Orthologue) score of 99.38%, and an Long Terminal Repeat Assembly Index score of 13.53, collectively signal the achieved high assembly quality. A comprehensive annotation yielded a total of 154,114 genes, and the associated BUSCO score of 95.78% for the annotated sequences attests to their completeness. The number of predicted NLR (Nucleotide-Binding and Leucine-Rich-Repeat genes) was 2107 with a large representation of NBARC (for nucleotide binding domain shared by Apaf-1, certain R gene products, and CED-4) containing domains (99.85%). Further comparative analysis of the proposed annotation-based assembly with high-quality known potato genomes, showed a similar genome metrics with differences in total gene numbers related to the ploidy status. The genome assembly and annotation of DC presented in this study represent a valuable asset for comprehending potato genetics. This resource aids in targeted breeding initiatives and contributes to the creation of enhanced, resilient, and more productive potato varieties, particularly beneficial for countries in Latin America.

## Teaser

The Diacol Capiro potato cultivar dominates Colombian cultivation. This autotetraploid, highly heterozygous cultivar, part of the Andigenum group, adapts to diverse environments across altitudes from 1800 to 3200 meters. The study presents a high-quality chromosome-scale assembly (DC) spanning 2.369 Gb, with 48 pseudochromosomes, an N50 of 50.5 Mb, and a BUSCO score of 99.38%. Annotation identified 154,114 genes, including 2107 NLR genes. Comparative analysis with known genomes highlights its significance for advancing potato genetics and breeding in Latin America.

## Introduction

Potato (*Solanum tuberosum* L.) is the most important noncereal crop in the world with a global production of 462 million tons in 2019. In Colombia, it is a crucial staple for ensuring food security and represents the principal income source for approximately 100,000 farming families (González-Orozco *et al.* 2023; Manrique-Carpintero *et al.* 2023). The high consumption and nutritional value of potato as a food security crop in the current global food system has been widely recognized (Devaux *et al.* 2020). The breeding of this crop has arisen mainly from two genepools, the upland Andigenum group with Andean landraces and the lowland Chilotanum group with Chilean landraces, with the Andigenum group being the most widely grown (Spooner *et al.* 2007; Gavrilenko *et al.* 2013). “Andigenas” potatoes are the most important within the Andigenum group. They are adapted to tuberization under short days, are autotetraploid (2n=4x=48) with tetrasomic inheritance, and highly heterozygous (Spooner *et al.* 2007).

**Fig. 1.**
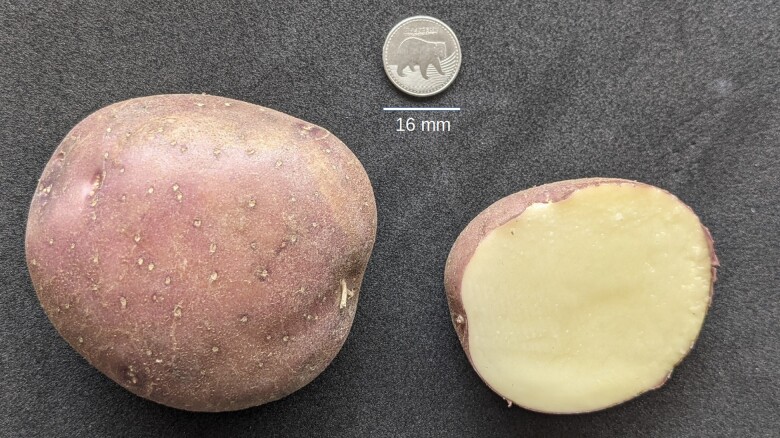
Tubers from cultivar Diacol Capiro.

Within the Andigenum group, the improved cultivar Diacol Capiro (also known as R12; Colombian Agricultural Institute (ICA) registration No. PAP-68-02), released in 1968, is one of the most widely cultivated in Colombia (SIPSA 2013; Romero *et al.* 2017). This variety stands out for its excellent quality for the potato flakes and sticks industry, and it also has good culinary quality (Porras Rodríguez and Herrera Heredia 2015). Moreover, it has been cultivated for several years, and it is adapted to a wide variety of regions from 1,800 to 3,200 meters above sea level in Colombia and in Ecuador, where it is distributed in the northern and central areas (Torres *et al.* 2011; Porras Rodríguez and Herrera Heredia 2015). This red tuber skin and cream flesh cultivar (Fig. 1) is tolerant to bacterial wilt (caused by *Ralstonia solanacearum*) and some viruses; though is susceptible to late blight (caused by *Phytophthora infestans*), powdery scab (caused by *Spongospora subterranea*) as well as other biotic and abiotic stresses (Andrade-Piedra and Torres 2011; Porras Rodríguez and Herrera Heredia 2015; Romero *et al.* 2017). Therefore, its genome sequence can contribute to shed light on molecular mechanisms to improve tolerance to these traits.

The knowledge of the potato genome opens great opportunities for further conservation and breeding efforts. Potato has been a species of great interest for researchers worldwide, as they strive to better understand the complexity of its genome in order to unlock the potential of new breeding techniques (Ghislain and Douches 2020). Since the publication of the first potato genome for the double monoploid DM1–3 516 R44 (PGSC 2011), several additional genomes have been published including the genome sequence of wild species such as *Solanum commersonii* (Aversano *et al.* 2015), *Solanum stenotomum* (Yan *et al.* 2021), and the M6 inbred clone of *Solanum chacoense* (Leisner *et al.* 2018). Moreover, several native Peruvian and Chilean landraces genomes were published including two from the Andigenum group (Kyriakidou, Achakkagari, *et al.* 2020; Kyriakidou, Anglin, *et al.* 2020), the heterozygous diploid potato *S. tuberosum* group Tuberosum RH89-039-16 using a combination of multiple sequencing strategies (Zhou *et al.* 2020), and an updated version of the DM1–3 516 R44 (assembly DM v6.1) using Oxford Nanopore Technologies long reads coupled with proximity-by-ligation scaffolding (Hi-C) to yield a chromosome-scale assembly (Pham *et al.* 2020). A recently haplotype-resolved chromosome-scale genome of the autotetraploid cultivar Otava from the Tuberosum group (SASA 2010), was sequenced from pollen nuclei used for gamete binning (Campoy *et al.* 2020; Sun *et al.* 2022). Recently, Hoopes *et al.* (2022) presented tetraploid phased assemblies of six potato cultivars from North America and Europe. Moreover, Bao *et al.* (2022) reported a haplotype resolved genome assembly of the tetraploid potato C88, cultivated in Asian countries. More assembled genomes from different genepools using new sequencing technologies and genome assembly algorithms are highly desirable to improve our understanding of the potato genome. These genome sequences are fundamental for the enrichment of the pan-genome of the tetraploid potato, first defined by Hoopes *et al.* (2022). This is considered a complex task due to the extraordinary diversity of the potato germplasm (Ghislain and Douches 2020).

The present study aims at achieving a chromosome-scale genome assembly into 48 pseudochromosomes for the tetraploid cv. Diacol Capiro (DC assembly) obtained by combining long reads from the PacBio Circular Consensus Sequencing (CCS) and Hi-C scaffolding data. The DC assembly is the first chromosome-scale assembly of a variety within the Andigenum group cultivated in the Andean region of Colombia and Ecuador. The genome assembly quality was compared to currently available potato chromosome-scale assemblies that include the monoploid DM v6.1 (Pham *et al.* 2020) and the haplotype diploid genome RH39-039-16 (Zhou *et al.* 2020). In addition, the DC assembly quality was compared against two scaffold level assemblies from the Andigenum group ADG1-CIP 700921 and ADG2-CIP 702853 (Kyriakidou, Anglin, *et al.* 2020). Moreover, annotation of the DC assembly was achieved and compared with six other high-quality potato annotations publicly available on SpudDB. Further analyses of Nucleotide-Binding and Leucine-Rich-Repeat (NLR) gene content and domain distribution was also performed.

## Materials and methods

### Plant material

Diacol Capiro tubers (Fig. 1) were planted under greenhouse conditions at the Tibaitata Research Center of the Corporacion Colombiana de Investigacion Agropecuaria (AGROSAVIA) using solarized soil mixed with peat in a 3:1 ratio in 20-kilogram pots with a temperature ranging from 19 to 27°C. The material was supplied by an extension program from AGROSAVIA, known as The Seed Plan that provides materials to farmers. Internodes containing lateral meristems obtained from stems of one plant were introduced under in vitro conditions. These were seeded independently in Murashige and Skoog (MS) medium (Murashige and Skoog 1962) supplemented with basal salts MS, vitamins, 3% sucrose, and 0.7% phytoagar and placed in growth chamber at 16–20°C and 6-h-light/8-h-dark photoperiod for four weeks. From the shoots obtained, different explants were seeded in independent tubes and placed in growth chamber for six weeks. Fifty-five in vitro plantlets were hardened for 20 days and then sown in a mixture of carbon slag:burned husk in a 1:1 ratio with weekly fertilization–irrigation under greenhouse conditions.

### DNA extraction and library preparation for sequencing

Before the start of flowering, about eight weeks after hardening, 100 grams of young leaf tissue that had not yet fully extended leaflets were collected and immediately frozen in liquid nitrogen and then stored at $-80^{\circ }$C. The tissue was sent for high-molecular-weight (HMW) genomic DNA extraction and HiFi library construction for PacBio CCS sequencing of three CCS cells to the Arizona Genomics Institute (Tucson, Arizona, USA). In addition, one gram of the same leaf tissue was subjected to High-throughput chromatin conformation capture (Hi-C) library construction using the ProximoHi-C Plant Kit (Phase Genomics) and sequenced on the Illumina HiSeq4000 under paired-end 150 bp mode by Phase Genomics (Seattle, Washington, USA). Additionally, 100 mg were used for DNA extraction with the DNeasy Plant Mini Kit (QIAGEN) followed by library preparation with the Nextera DNA Flex Kit in Dual Index format (Illumina) that was sent for paired-end sequencing on the Illumina HiSeq4000 System to Macrogen (Seoul, South Korea). The HiFi data consisted of 5.3 M reads, N50 of 16.754 bases (b), and a total of 89 Gb sequenced. The Illumina dataset consisted of 1.49 G paired-end reads with a total of 226 Gb; these reads were not used in the assembly, they were used only for technical validation.

### Size, ploidy, and heterozygosity estimation

Estimates for the DC genome were obtained using the HiFi and Illumina data sets. For both datasets, KMC3 (Kokot *et al.* 2017) was used to obtain k-mer statistics, while GenomeScope 2.0 and Smudgeplot were used to obtain the genome profiling (Ranallo-Benavidez *et al.* 2020).

### De novo genome assembly

The HiFi reads were run into multiple de novo assemblers: HiCanu (Nurk *et al.* 2020), Hifiasm (Cheng *et al.* 2021), and IPA from PacBio (Biosciences 2020), since these algorithms are designed to assemble long HiFi reads. Moreover, the assembler Flye (Kolmogorov *et al.* 2019) was used because it has remarkable results for genome assembly using long reads (Reyes-Herrera *et al.* 2023). The best assembly was obtained with HiCanu based on the BUSCO (Benchmarking Universal Single-Copy Orthologue) 5.1.0 score (Manni *et al.* 2021) (using embryophyta_odb10 and solanales_odb10 lineages) as well as the metrics: N50, L50, total length, largest contig, and total contigs. These metrics were obtained by QUAST v5.0.2 (Gurevich *et al.* 2013) (see Table 1).

**Table 1.** Genome assembly statistics of HiFi reads.

Assembly	HiCanu	Flye	Hifiasm	IPA
N50	1,593,269	560,881	10,240,684	787,629
L50	370	747	52	352
Total Length	2,456,789,690	2,189,886,870	1,801,232,397	1,159,482,885
Largest Contig	24,389,854	12,742,642	34,911,349	10,321,014
Total Contigs	6,692	15,383	4,538	6,830
% Complete BUSCO embryophyta (n=1,614)	99.32	99.19	99.31	81.60
% Complete BUSCO solanales (n=5,950)	99.03	99.00	98.90	78.50

The HiFi and Illumina reads were used separately to estimate genome size, heterozygosity and to confirm ploidy (Supplementary Figs. S1–S3). From the HiFi reads, a haploid genome size of 679 Mb was estimated using GenomeScope (Ranallo-Benavidez *et al.* 2020). The parameters used were k-mer length = 21 and ploidy = 4, which corresponds to a total length of 2,716 Mb. The HiCanu assembly length of 2,456 Mb was the closest to the expected tetraploid assembly length (see Table 1).

For the scaffolding process, HiCExplorer (Ramírez *et al.* 2018) was used to filter the Hi-C data. As a result, 11.08% of the initial dataset were valid contacts. Despite this, 30% of the reads did not map uniquely because of polyploidy and repetitive regions that were filtered out by HiCExplorer. Nevertheless, over 40% of the paired reads were located more than 10 Kb apart and were confirmed to correspond to actual Hi-C junctions. Thus, two datasets were used: the valid contacts and the nonuniquely mapping reads (hereinafter referred to as raw Hi-C).

Then, we used both datasets and the ALLHiC pipeline for the scaffolding suitable for an autopolyploid genome (Zhang J *et al.* 2018; Zhang X *et al.* 2019). The ALLHiC pipeline involved five steps: pruning, partitioning, rescue, optimization, and building. At the pruning step, ALLHiC identified a set of allelic contigs based on synteny to the reference chromosome scale-assembled genomes DM v6.1 (Pham *et al.* 2020) and RH39-039-16 (Zhou *et al.* 2020). Additionally, ALLHiC provided a corrector to detect and fix misjoined contigs using Hi-C signals. Eight assemblies were generated by varying input data and parameters that included: the Hi-C data used for correction and scaffolding, the initial genome assembly, and the reference genomes at the pruning step (see Table 2). The filtered valid contacts and the raw Hi-C reads were used as input for the Hi-C data. Details regarding the data used for the scaffolding strategies can be found in Table 2.

**Table 2.** Data used for scaffolding strategies (st) in ALLHiC: (1) Initial Assembly, (2) Hi-C Data used for Genome Correction, (3) Corrected Assembly used for Scaffolding, (4) Hi-C Data used for Scaffolding and, (5) Reference Genome used for the Pruning step.

ALLHiC step	Genome Correction		Scaffolding		Pruning
Strategy	(1) Initial Assembly	(2) Hi-C Data	(3) Corrected Assembly	(4) Hi-C Data	(5) Reference Genome
st0	HiCanu	none	HiCanu	Hi-C raw	RH89-039-16
st1	HiCanu	Hi-C raw	HiCanu + Hi-C raw	Hi-C raw	RH89-039-16
**st2**	**HiCanu**	**Hi-C raw**	**HiCanu + Hi-C raw**	**Hi-C valid contacts**	**RH89-039-16**
**st3**	**HiCanu**	**none**	**HiCanu**	**Hi-C valid contacts**	**RH89-039-16**
st4	HiCanu	Hi-C valid contacts	HICanu + Hi-C valid contacts	Hi-C valid contacts	RH89-039-16
st5	HiCanu	none	HiCanu	Hi-C valid contacts	DMv6.1
**st6**	**HiCanu**	**Hi-C raw**	**HiCanu + Hi-C raw**	**Hi-C valid contacts**	**DMv6.1**
st7	HiCanu	Hi-C valid contacts	HICanu + Hi-C valid contacts	Hi-C valid contacts	DMv6.1

The selected strategies are highlighted in bold.

**Fig. 2.**
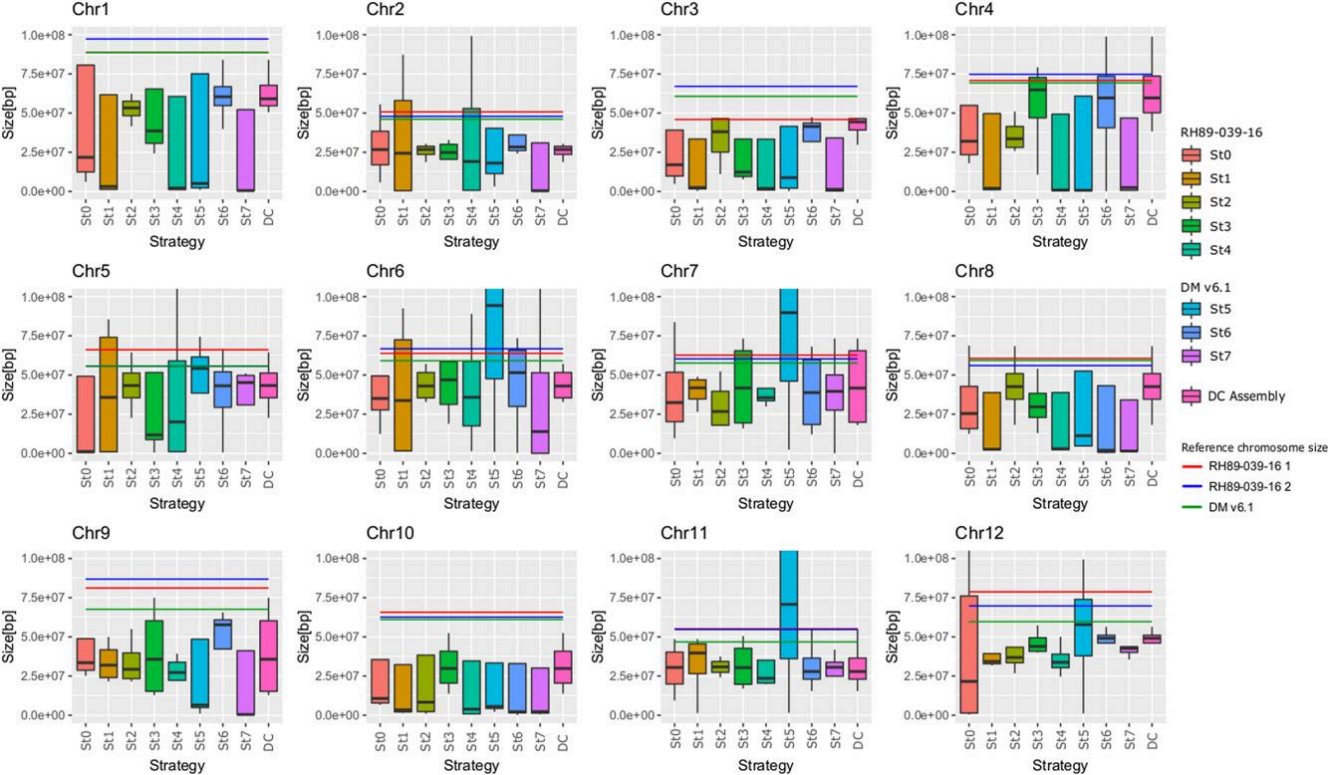
Boxplots displaying the range of sizes of the four largest scaffolds for each of the 12 chromosomes (Chr) using different assembly strategies (st0–st7). The DC assembly is also included. In the legend, the strategies are separated based on the reference genome used at the pruning step. The horizontal lines above indicate the size of each chromosome in the reference genomes DMv6.1 (green) and RH89-039-16 with two haplotypes (blue and red).

Most of the resultant assemblies had 12 homologous groups with sets of large scaffolds plus a set of additional contigs. For each homologous group, the differences in size were plotted for the four largest scaffolds (Fig. 2). None of the assemblies exhibited clear superiority over the others, for this reason a hybrid strategy was explored. Three assembly strategies were selected (st2, st3, and st6), resulting in sets of four scaffolds with similar sizes by most chromosomes. This was reflected by the lowest standard deviation of the scaffold size with equal BUSCO scores (Supplementary Table S1).

**Fig. 3.**
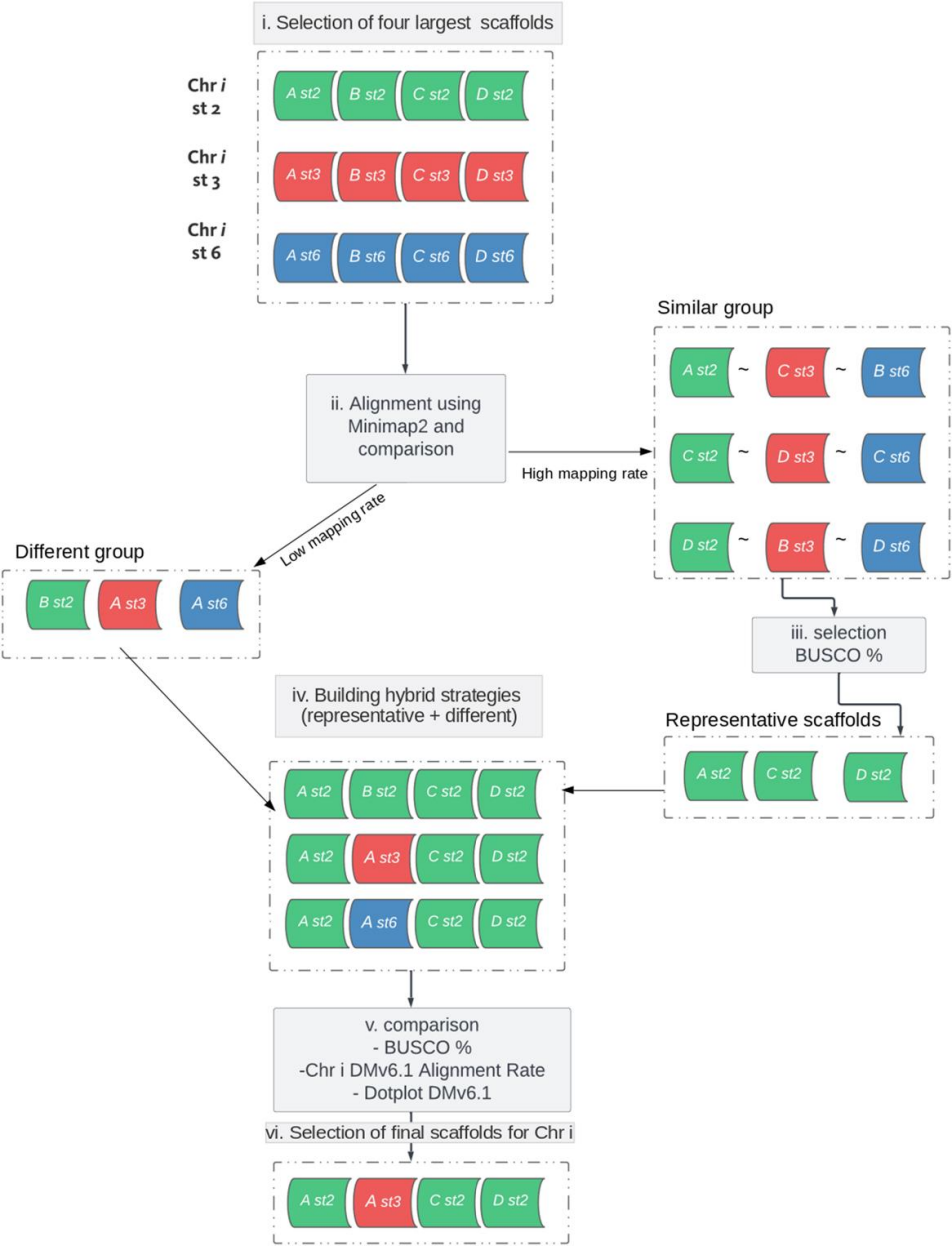
Pipeline for the selection of pseudo-chromosomes using the results from three strategies. (i) Selection of the four largest scaffolds (A, B, C, and D) for each chromosome (Chr*i*) for the strategies st2, st3, and st6. (ii) Alignment with Minimap2 among the scaffolds of each strategy and comparison to identify similar groups and different scaffolds. (iii) Identification of the representative scaffolds, between similar scaffolds, based on BUSCO score. For this example $A_{st2}$, $C_{st2}$, and $D_{st2}$ had the highest BUSCO score. (iv) Building hybrid strategies combining representative scaffolds and the different group, each row is an alternative combination of scaffolds to represent the Chr*i*. (v) Comparisons of hybrid strategies based on the BUSCO score, median alignment rate to DMv6.1 and contiguity based on dot plot with DMv6.1. (vi) Final selection of best candidates as pseudo-chromosomes for Chr*i*, in this case $A_{st2}A_{st3}C_{st2}D_{st2}$ is a combination of scaffolds from st2 and st3.

Then, the variations between the selected assembly strategies (st2, st3 and st6) were examined using dotplots to the reference DMv6.1 (Supplementary Figs. S4–S6). Nevertheless, no single strategy has emerged as superior to the others, as indicated by the mapping rate and QUAST statistics outlined in Table 3. The assembly and scaffolding steps were not deterministic; each strategy produced a different output. To achieve the best possible genome assembly, these outputs were evaluated to construct a hybrid assembly, which comprises the best possible set of pseudo-chromosomes for each homologous group. For this purpose, the four largest scaffolds were compared for the three strategies for each chromosome, and a hybrid configuration (combining scaffolds from st2, st3, and st6) was obtained (see the pipeline of Fig. 3). The Minimap2 software (Li H 2018) was used to align the scaffolds among the three strategies (st2, st3, and st6) at a chromosome-scale with the identification of similar scaffolds (step ii, Fig. 3). Then, hybrid sets were built using one scaffold selected among the similar groups and the different group (steps iii and iv, Fig. 3). The best set was selected based on three criteria (see steps v and vi, Fig. 3): (1) the BUSCO score for embryophyta_odb10 (as shown in Supplementary Figs. S7–S18); (2) the median alignment rate to DM v6.1 (as shown in Supplementary Fig. S19); and (3) the dotplots to DM v6.1 (as shown in Supplementary Figs. S4–S6). The procedure to build hybrid chromosomes was successful for chromosomes 1, 3, 4, and 7. For all other chromosomes, scaffolds from only one of the strategies (st2, st3, or st6) was the best configuration following the three criteria (see details for selection in the Supplementary Table S2). Table 3 shows the comparison results of the three selected assembly strategies (st2, st3, st6) and the resultant assembly named DC.

**Table 3.** Comparisons of the assembly strategies st2, st3, and st6 with the DC assembly.

Assembly Metrics		st2	st3	st6	DC
QUAST	N50	38,325,480	50,491,741	**59,274,238**	50,589,760
	L50	21	**16**	13	19
	Total Length	2,176,301,816	2,313,398,270	2,052,867,247	**2,369,577,969**
	Largest Contig	76,220,365	170,413,983	163,405,974	98,785,543
	Total Contigs	1,437	1,481	**1,133**	1,194
Mapping Rate	% Illumina Mapping Rate	98.74	**99.23**	98.30	98.58
Assembly Quality	LTR Assembly Index (LAI)	12.65	13.14	13.43	**13.53**

The best metrics per line are highlighted in bold.

After the scaffolding with ALLHIC, a difference of approximately 370 Mbp in length was spotted between the initial Canu assembly and the DC assembly. A difference in length attributed to the scaffolding was expected. However, ALLHIC did not include contigs with weak Hi-C signals, and even though the pipeline had a rescue step, 15% of contigs remain unrecovered. Lost contigs were recovered in the ALLHIC scaffolding and were checked for alignments against the DC assembly. A contig coverage was defined as the portion of the contig aligned divided by the contig length. The recovered contigs that had a contig coverage lower than the median contig coverage distribution were added to the assembly. In addition, Juicebox (Durand *et al.* 2016) was used to check and fix the corresponding heatmap for each chromosome manually (Supplementary Fig. S20).

### Contaminant screening

Blobtools v2 pipeline (Challis *et al.* 2020) was used to screen contaminants and to perform a quality assessment for the DC assembly. This pipeline uses assembly coverage, as well as BUSCO, Blast, and DIAMOND v2.0.11 (Buchfink *et al.* 2021) results. Two contigs were identified as Proteobacteria and removed from the assembly.

### Assessment of the DC assembly

First, the Illumina dataset was mapped to the assembly to assess completeness and accuracy using BWA-MEM v0.7.12 (Li H 2013) and SAMtools v1.8 (Li H *et al.* 2009) was used to obtain alignment statistics. Second, BUSCO v5.1.0 (Manni *et al.* 2021) was used to assess the completeness of the gene space for two lineages: the embryophyta_odb10 and the solanales_odb10. Third, the Long Terminal Repeats (LTR) Assembly Index (LAI) was used to evaluate the assembly continuity using the LTRharvest v1.6.1 (Ellinghaus *et al.* 2008), LTR_FINDER_parallel v1.1, LTR_retriever v2.9.0 (Ou and Jiang 2017), and LAI (Ou *et al.* 2018). Fourth, Meryl was used to count the k-mers and Merqury (Rhie *et al.* 2020) was used to perform a reference-free evaluation. This approach involved comparing the assembly to un-assembled reads based on k-mers. Fifth, the DC assembly was compared against the Andigenum group assemblies, ADG1 and ADG2 (Kyriakidou, Anglin, *et al.* 2020). Sixth, the genome assembly quality was compared with two chromosome-scale assemblies currently available for potato: (1) the doubled monoploid assembly DMv6.1 from the *S. tuberosum* group Phureja that corresponds to an updated version (Pham *et al.* 2020) from the first version of the potato genome (PGSC 2011) and, (2) a haplotype-resolved assembly for a diploid potato from the Tuberosum group (Zhou *et al.* 2020).

### Annotation

A repeat library was constructed using RepeatModeler v1.0.8 (Smit and Hubley 2008). The repeat library was screened using ProtExcluder v1.1 (Campbell *et al.* 2014) in order to exclude existing proteins. The obtained repeat library was used to mask the tetraploid potato genome. Protein coding genes were predicted on the potato-masked genome. Maker v3.01.02 (Cantarel *et al.* 2008) annotation pipeline was used to produce consensus gene models. Different resources such as ab-initio gene prediction, homology search, and RNA-seq were provided in an iterative process to Maker. Viridiplantae reviewed proteins from Uniprot were used as homology evidence along with DMv6.1 proteins (PGSC 2011) and tomato ITAG4.0 proteins (Hosmani *et al.* 2019). Public available RNAseq data from SRA (Supplementary Table S3) from different life stages were mapped to the genome using HiSAT2 v2.1.0 (Kim *et al.* 2015) and provided as evidence. There was a constraint that hindered the identification of specific genes unique to the DC cultivar, due to the absence of RNA-Seq data specific to this cultivar. Nonetheless, the majority of the RNA-seq samples analyzed corresponded to the *S. tuberosum* Andigenum group (Lin *et al.* 2015; Ponce *et al.* 2022; Bozan *et al.* 2023), to which the DC cultivar belongs.

Additionally, ab-initio gene prediction was obtained from Augustus (3.4.0) (Stanke *et al.* 2008) through the pipeline BRAKER v2.1.4 (Hoff *et al.* 2019). The final consensus gene models set resulted from three rounds of Maker using previous inputs. BUSCO was used to assess the completeness of the gene prediction. Functional annotation of the predicted protein sequences was achieved using Eggnog software (Buchfink *et al.* 2021; Cantalapiedra *et al.* 2021) that aligned protein sequences against public databases, including SwissProt, TrEMBLE and KEGG, and eggNOG orthology data (Huerta-Cepas *et al.* 2019). Additional values were obtained for InterPro and Gene Ontology (GO) databases by InterProScan (Quevillon *et al.* 2005).

The NLR-Annotator tool (Steuernagel *et al.* 2020) was employed to identify candidate Nucleotide-Binding and Leucine-Rich-Repeat (NLR) domains. This tool runs an in silico analysis of the annotated regions of the DC Assembly. The same approach was used to analyze annotations of six assemblies available in the SpudDB database (http://spuddb.uga.edu/index.shtml). These include three tetraploid assemblies: Atlantic (Atlantic_v3 annotation) (Hoopes *et al.* 2022), Cooperation-88 (C88.v1 annotation) (Bao *et al.* 2022), and Otava (Otava.v1 annotation) (Sun *et al.* 2022). In addition, one diploid assembly RH89-039-16 (Zhou *et al.* 2020), and two monoploid assemblies DMv6.1 (Pham *et al.* 2020) and M6 (M6_v5 annotation) (Leisner *et al.* 2018) were included.

## Results and discussion

### Size, ploidy, and heterozygosity estimation

HiFi and Illumina data sets were used to estimate size, ploidy and heterozygosity in the DC genome. For the HiFi dataset, an estimated coverage of 100x was obtained, while for the Illumina dataset, an estimated coverage over 300x was obtained. The estimated genome haploid length was 682 Mb and 679 Mb for the Illumina and HiFi datasets, respectively (a 0.43% difference between both estimated lengths, see Supplementary Figs. S1–S2). Considering a genome size of 679 Mb for the haploid genome and 2.716 Gb for the tetraploid genome, the DC assembly covered 87.5 % of the estimated length. The repeat length estimation for each haploid genome ranged from 296 to 300 Mb for the Illumina dataset. Smudgeplot (Ranallo-Benavidez *et al.* 2020) confirmed that the DC assembly was from a tetraploid genome for both datasets (see Supplementary Fig. S3). The estimated heterozygosity ranged from 4.13% to 6.78% for the Illumina dataset and from 3.88% to 5.82% for the HiFi dataset. This falls within a similar range to the previously reported tetraploid ADG1 (3.52%) and ADG2 (7.75%) Andigenum assemblies (Kyriakidou, Anglin, *et al.* 2020).

### DC assembly evaluation

**Fig. 4.**
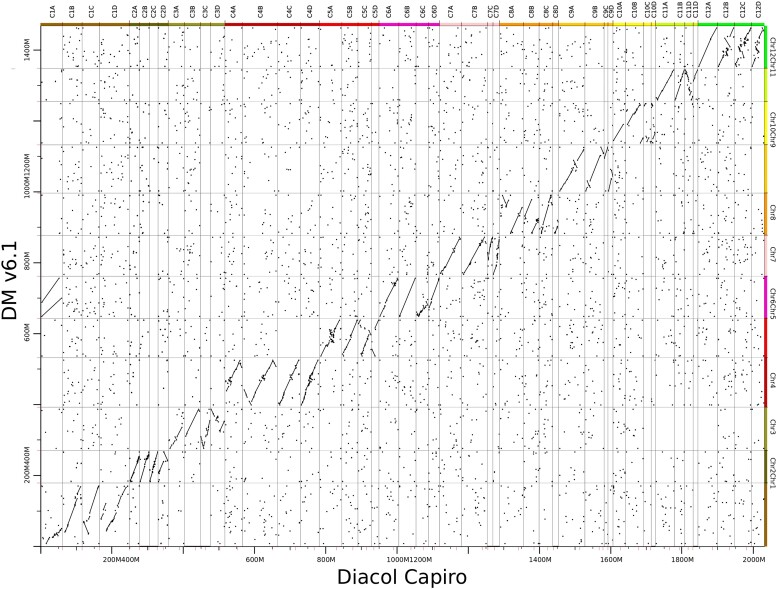
Dotplot with the alignment of DC (Diacol Capiro) assembly against the DMv6.1 chromosome-scale assembly. Sets of 4 homologous chromosomes aligned to each DMv6.1 chromosome are observed.

The final DC assembly had a total length of 2,369,577,969 bases (Tables 3 and 4), with 88.26% of these bases being anchored to 48 pseudo-chromosomes (see dotplot for the assembly in Fig. 4). The tetraploid assembly has 1,194 contigs (99.9% of the contigs longer than 10 Kb).

**Table 4.** Genome assembly statistics for *S. tuberosum* chromosome-scale assemblies used as references and *S. tuberosum* Andigenum group assemblies.

*S. tuberosum Group*	*Tuberosum*	*Phureja*	*Andigenum*		
Genome Assembly	RH89-039-16	DMv6,1	ADG1	ADG2	DC(1194)
Sequencing Technology	10xG + ONT + CCS + Hi-C	ONT+Hi-C	Illumina + PacBio RS II	Illumina	Pacbio HiFi+Hi-C
N50	66,116,962	59,670,755	121,761	4,940	50,589,760
L50	11	6	1,313	54,927	19
Total Size	1,673,555,276	741,585,035	841,412,671	947,924,978	2,369,577,969
Largest Contig	97,287,411	88,591,686	3,383,905	102,027	98,785,543
Total Contigs	3,024	288	35,955	248,063	1,194
Illumina Mapping Rate (reads properly paired)	99.90	98.05	85.70	86.68	98.58
% Complete BUSCO Embriophyta (n=1,614)	99.32	99.38	85.63	48.69	99.38
% Complete and Duplicated BUSCO Embriophyta (n=1,614)	68.22	1.92	7.68	5.63	87.79
% Complete BUSCO Solanales (n=5,950)	98.87	98.67	87.08	56.21	98.96
% Complete and Duplicated BUSCO Solanales (n=5,950)	69.98	2.32	9.53	6.48	89.26
LAI	6.47	13.56	9.06	7.29	13.53
% Genome Fraction RH89-039-16	100	35.37	33.35	28.27	57.75
% Genome Fraction DMv6	81.26	100	72.71	68.38	85.66
% Genome Fraction ADG1	73.24	68.59	100	53.70	80.69

A 98.58% of the illumina reads were aligned and properly paired to the DC assembly. The embryophyta_odb10 lineage comprised 1,614 BUSCO orthologs, of which 1,604 were complete in the DC assembly (representing 99.38%) (Table 4). These contained 186 single copies and 1,418 duplicated copies. The remaining orthologs for this lineage were five fragmented and five missing. The solanales_odb10 lineage comprised 5,950 BUSCO orthologs, of which 5,888 were complete in the DC assembly representing 98.96%, 577 single copy, and 5,311 duplicated, while five of the remaining orthologs were fragmented, and 57 were missing. The assembly was constructed utilizing a hybrid strategy, which involved the fusion of scaffolds from different assemblies for four specific chromosomes. The primary objective was to extract the most optimal assembly achievable from the available dataset. Notably, the DC assembly exhibited superior performance compared to the input assemblies in several key metrics, including quality (as measured by LAI), N50, and total length. In all other metrics, the DC assembly’s performance was similar to that of the original assemblies but never demonstrated inferior performance (Table 3). While the hybrid strategy proved effective, it is important to note that further enhancements in assembly quality require the inclusion of additional data.

**Fig. 5.**
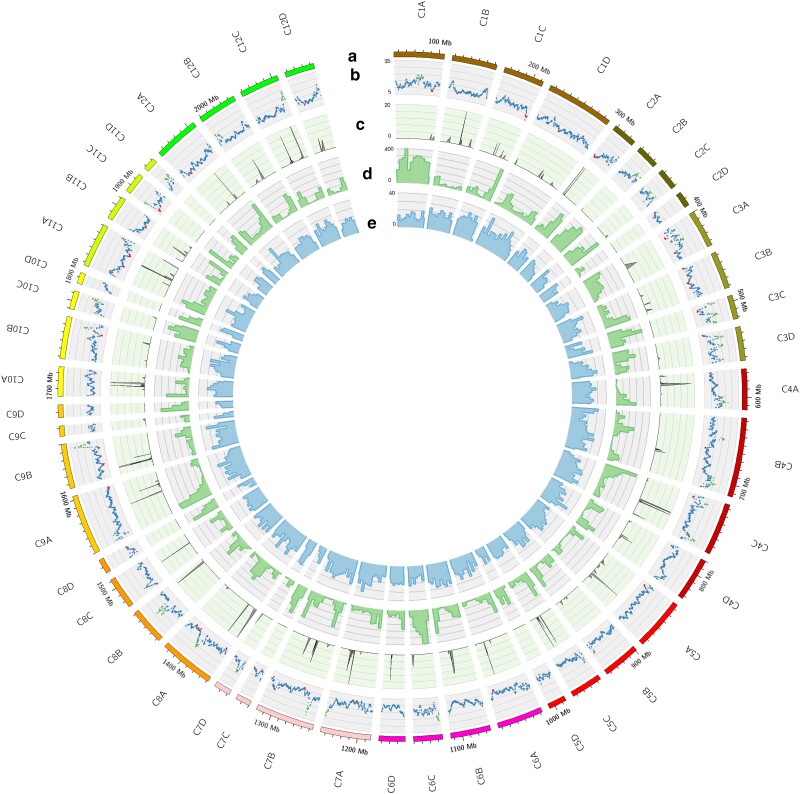
Circos plot for the DC assembly. a) 48 pseudo-chromosomes. b) LAI over 100 kb sliding windows. c) Reads from CENH3-ChIP-seq (Gong *et al.* 2012) aligned to the DC assembly, with chromosomes divided into 100 kb windows. The resultant peaks detected in this analysis are indicative of potential centromeric regions within the genome. d) Histogram for genes using a window size of 5 Mb. e) Histogram for LTRs with a window size of 5 Mb.

Figure 5 illustrates five concentric circles, each providing specific insights regarding the scaffolds from the DC assembly. The circle a encompasses the depiction of the 48 pseudo-chromosomes. The circle b showcases a scatterplot portraying the LAI. Notably, the DC assembly exhibits an LAI score of 13.53, fitting reference genomes of high quality, i.e. DMv6.1 (Table 4). LAI scores ranging from 10 to 20 are actually indicative of high quality. The assembly predominantly displays an LAI falling within the 10< LAI <20 range, with notable peaks even surpassing an LAI of 20. The circle c presents the alignment of reads from CENH3-ChIP-seq (Gong *et al.* 2012) to the DC assembly, indicative of centromeres. Among the 30 pseudo-chromosomes, a distinct peak is evident, which serves as an indicator of the centromeres. However, for the remaining pseudo-chromosomes, there is an absence of such a peak, potentially indicating the presence of distinct centromeric regions. This agrees with observations of the C88 tetraploid genome assembly (Bao *et al.* 2022). The last two circles (d, e) depict the gene and LTR (Long Terminal Repeat) distribution within 5 Mb windows. As expected, there is a noticeable enrichment of genes beyond the centromeres or in proximity to the telomeres and a corresponding reduction around the centromeres consistent with the gene density previously reported (Bao *et al.* 2022; Hoopes *et al.* 2022). In contrast, the distribution of LTRs appears to be more uniformly spread.

At last, a reference-free assessment using Merqury for the DC assembly resulted in a high accuracy consensus Quality Value (QV) of 65.458, and k-mer completeness of 92.39%.

### Comparison of the DC genome assembly to the ADG1, ADG2 Andigenum group and other tetraploid assemblies

Table 4 shows genome statistics for the genome assemblies ADG1, ADG2, and DC assembly. The ADG2 assembly was built only on Illumina reads, the ADG1 assembly used Illumina and PacBio RS II reads, and the DC assembly from this study used PacBio HiFi to build the initial assembly along with Hi-C data for the scaffolding. By including long PacBio HiFi reads, the number of contigs was reduced significantly and the N50 increased (Table 4). The BUSCO score increased as the genome is less fragmented. For the embryophyta_odb10 lineage, the BUSCO score was 85.63%, 48.69% and 99.38% for the ADG1, ADG2, and DC assemblies, respectively. However, the complete and duplicated BUSCO score (embryophyta_odb10) was significantly higher for the DC assembly with 87.79% indicating that it recovered more than one haplotype as compared to the 7.68% for the ADG1 and 5.63% for the ADG2. In addition, the DC assembly had the best LAI score (13.53), which corresponds to reference genomes of high quality (between 10 and 20), while the ADG1 and ADG2 had LAI scores of 9.06 and 7.29, which correspond to genomes of draft assembly quality. The repetitive portion was lower in the DC genome with 49.92% compared to the 60.2% in ADG1 (Kyriakidou, Anglin, *et al.* 2020), and the 65–68% of seven tetraploid genome assemblies (Hoopes *et al.* 2022; Sun *et al.* 2022). The repetitive sequences present in the DC genome assembly led to the masking of 44% of the assembly. The DC assembly repetitive portion was closer to the range reported in a previous comparative analysis of repetitive sequences in potato and tomato species (Gaiero et al. 2019). The predominant classes of transposable elements (TEs) were retrotransposons (37%) and DNA transposons (5%) (refer to Supplementary Fig. S21). In comparison to six high-quality tetraploid genome assemblies, these two classes were similarly found to be the most abundant TEs, constituting 26% and 1.48%, respectively (Hoopes *et al.* 2022). This observation aligns with findings from the potato super pangenome study involving 296 diploids and polyploids, where retrotransposons were identified as the predominant TE class (Bozan *et al.* 2023). The lengths or base pair sizes of LTRs, DNA transposons, and small RNAs in DC fell within a comparable range to those observed in the super pangenome (see Supplementary Fig. S16 in Bozan *et al.* 2023, whereas LINEs and simple repeats were comparable to those reported in the previously mentioned tetraploid assemblies (refer to Table S9 in Hoopes *et al.* 2022).

### Comparison of the DC genome assembly to chromosome-scale assemblies DMv6.1 and RH89-039-16

Genome assembly statistics for these genomes are shown in Table 4 . The number of contigs for the assemblies was 288 for DMv6.1 (a monoploid genome), 3,024 for RH89-039-16 (a diploid genome), and 1,194 for DC (a tetraploid genome) with the contig number for the tetraploid being about four times that of the monoploid as most likely expected. The N50 was 59 Mb for DMv6.1, 66 Mb for RH89-039-16, and 50 Mb for DC. However, in the complete BUSCO score, the three genomes have similar scores for the embryophyta_odb10 lineage with 99.38% for DMv6.1, 99.32% for RH89-039-16, and 99.38% for DC. The BUSCO score indicated completeness and duplication, with the percentage of duplication increasing proportionally with the ploidy level, as anticipated. Thus, for the monoploid DMv6.1 the score was 1.92 %, for the diploid RH89-039-16 the score was 68.22% and for the DC was 87.79%. For the solanales_odb lineage, the complete BUSCO score was also similar with 98.67%, 98.87%, and 98.96% for the DMv6.1, RH89-039-16, and DC, respectively. The LAI index that assesses the genome assembly quality was 6.47 for the RH89-039-16, which corresponded to the draft genome quality. In contrast, 13.56 and 13.53 for the DMv6.1 and DC assemblies, respectively, both fell in the reference genome category. An extended version of this comparative analysis, including two additional assemblies Solyntus (van Lieshout *et al.* 2020) and M6 (Leisner *et al.* 2018) is in the Supplementary Table S4.

### Annotation

A total of 154,114 gene predictions were identified, 86.16% of which were anchored to the 48 pseudo-chromosomes (see Supplementary Fig. S22 for a detailed distribution by chromosome). The DC assembly annotated sequences had a BUSCO score of 95.78% for the embryophyta_odb10 lineage mode transcriptome (see Table 5). The range of BUSCO scores for other six published high-quality assembly annotations was between 90.08% for M6_v5 (Leisner *et al.* 2018) and 99.25% for C88.v1 (Bao *et al.* 2022). This places the DC annotation well within the range of quality of the assemblies in terms of BUSCO score. Thus, a high-quality DC annotation can be inferred from these comparisons. This is noteworthy considering that previously annotated tetraploids derived from phased genome assemblies used genome sequence coupled with RNA sequence data (Bao *et al.* 2022; Hoopes *et al.* 2022; Sun *et al.* 2022), S1 mapping (Bao *et al.* 2022), or single-cell pollen sequence (Sun *et al.* 2022)—all of which facilitates annotation derived genome assembly of autotetraploid potatoes. The approach used in DC leverages available high-quality annotation data, which results in comparable BUSCO metrics that indicates completeness.

**Table 5.** A comparison of potato genome assembly annotations, arranged alphabetically, was conducted based on multiple factors including BUSCO score (using the mode transcriptome and lineage embryophyta_odb10), the number of genes, and the presence of NLR putative genes.

Assembly	Annotation	Citation	BUSCO v4.1.2	Number of Genes	NLR Genes
Atlantic	Atlantic v3.0 High Confidence Gene Model Set	(Hoopes *et al.* 2022)	95.53	269,097	2,186
Cooperation-88 (C88)	C88 v1.0	(Bao *et al.* 2022)	99.25	150,853	4,065
**DC**	**DC v1.0**	This publication	**95.78**	**154,114**	**2,107**
DMv6.1	DM 1-3 516 R44 - High Confidence Gene Model Set - v6.1	(Pham *et al.* 2020)	92.87	32,917	566
M6	M6_v5.0 High Confidence Gene Model Set	(Leisner *et al.* 2018)	90.08	76,300	1,148
Otava	Otava v1.0	(Sun *et al.* 2022)	97.08	154,672	2,937
RH89-039-16	RH89-039-16 v3.0 Transcript sequences (cDNA) of gene models in diploid potato	(Zhou *et al.* 2020)	97.33	76,394	378

The DC annotation is highlighted in bold.

Regarding the number of predicted genes, this ranged from 32,917 for the monoploid DMv6.1 to 269,097 for the tetraploid Atlantic. The DC number of genes was more similar to the tetraploid C88 (154,114 vs. 150,853). The cultivar C88 has a pedigree from *S. tuberosum* group Andigena as the paternal plant and an Indian potato as the maternal source (Bao *et al.* 2022), while DC is from the same group Andigena. Thus, similarities exist in gene content between these two varieties: 95.49% of C88 predicted genes aligned to the DC annotation. Moreover, 89.13% of the DC predicted genes aligned to the C88 annotation with a percentage of identical positions higher than 70%. While both annotations share most of the genes, differences can be attributed to unique specificities inherent to each variety. In general, the number of genes increased with the ploidy as anticipated, since any ploidy reduction would lead to gene loss (Sun *et al.* 2022), and tetraploid potato would provide more gene copies as backups for defective alleles (Bao *et al.* 2022).

Predicted NLR genes were also identified in silico in DC and the six other assemblies (Table 5). The number of candidate NLRs was 2,107 for DC and 2,186, 4,065 and 2,937 for the tetraploids Atlantic, C88, and Otava, respectively. The disparities in numbers, as compared to those reported for C88 (2,262 NLR genes) in Bao *et al.* (2022), may arise from the utilization of distinct methodologies in both analysis. The C88 used RGAugury pipeline (Jupe *et al.* 2012; Li H *et al.* 2016) while DC used NLR-Annotator (Steuernagel *et al.* 2020). In addition, the M6_v5 showed a greater abundance of NLRs (1,148) as compared to DMv6.1 (566) and RH89-039-16 (378) assemblies. Thus, the analysis revealed a higher number of predicted NLR genes in the four tetraploids (>2,000 genes) followed by the diploids and the monoploid assemblies, indicating a promising gene reservoir to tackle innate immune responses.

**Fig. 6.**
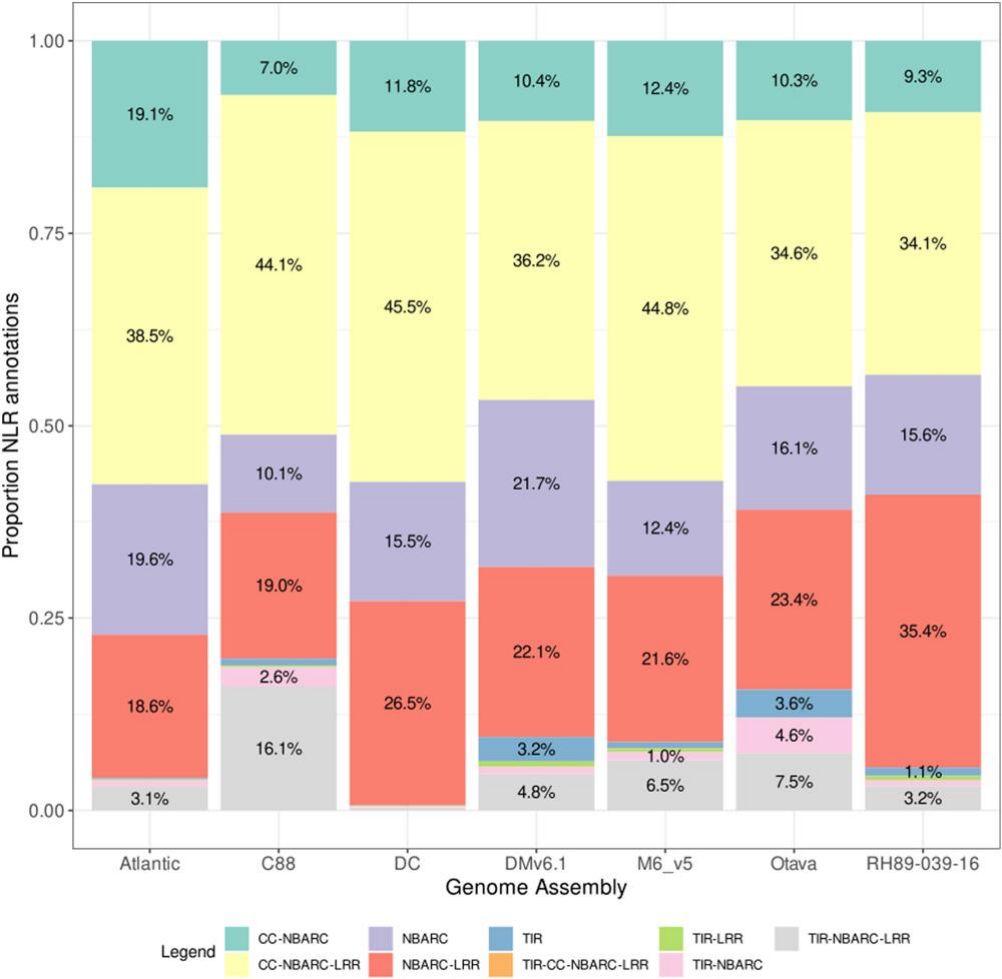
Proportions of predicted NLR (Nucleotide-binding Leucine-rich Repeat) genes across six distinct potato genome assemblies. The representation of various combinations of domains within NLR genes is visualized using different colors (see legend). Percentages below 1% were not included in the figure to prevent visual overlaps and maintain clarity.

NLR domain genes belong to one of the largest multigene families in plants (Ercolano *et al.* 2022). The largest class of plant resistance (R) genes encodes for a conserved NBARC (for nucleotide binding domain shared by Apaf-1, certain R gene products, and CED-4) fused to leucine-rich repeats (NBARC-LRR proteins), sometimes in proximity with other elements such as TIR (Toll/Interleukin-1 Receptor) domains (Dangl and Jones 2001). The proportion for various of these combinations of domains within NLR genes is visualized using different colors in Fig. 6. Four main domain combinations of NLR genes are consistently prominent across all seven assemblies: CC-NBARC (coiled-coil domain-NBARC), CC-NBARC-LRR, NBARC, and NBARC-LRR. Each of these configurations represents more than 7% of the total.

The TIR-NBARC-LRR combination is also observed in all seven assemblies. Among these, C88 exhibits the largest proportion of this domain combination (16.1%), while DC shows the smallest (0.33%) (Fig. 6). The presence of the other four domain combinations involving TIR (TIR, TIR-CC-NBARC-LRR, TIR-LRR, TIR-NBARC) is diminished in most assemblies. The sum of proportions of these four combinations per assembly range from 0.66% in DC to 8.23% in Otava. Thus, DC, a variety from the Andigena group known to be susceptible to late blight and other pathogens, holds a modest proportion of TIR domain combinations. In contrast, C88, a late blight-resistant variety from the same group (Bao *et al.* 2022), exhibits an important proportion of the predicted TIR domain combinations. The lowest TIR domain content in DC or other distinctive features might render it more susceptible, offering opportunities for improvement through new introgressions. In fact, C88, which carries the late blight resistance R1 (a CC-NBS-LRR (Ballvora *et al.* 2002)) and R2 gene clusters, has been shown to contain wild introgressions within NLR genes, potentially explaining the origin of functional resistance genes (Bao *et al.* 2022). Wild introgressions were also observed in the first tetraploid potato pangenome, which included the Atlantic variety containing the R1 cluster. This thorough examination of disease-related genes in DC will be crucial for employing gene-editing techniques to enhance the cultivar (Tiwari *et al.* 2022).

Functional annotation was also successfully acquired for 143,486 gene predictions within the DC assembly, constituting 93.1% of the total identified genes. Among these functional annotations, the seed orthologs distribution is as follows: 61.7% for *S. tuberosum*, 23.9% for *Solanum lycopersicum*, 4.51% for *Nicotiana tormentosis*, and 4.33% for *Nicotiana sylvestris*. These identified orthologs collectively account for 94.5 % of the functional annotations, and as expected, are mainly represented by the phylogenetically related *Solanum* species. At last, we used phylostratr (Arendsee *et al.* 2019) to estimate the phylostratum of every gene in the DC annotation and obtained a total of 5,349 of genes specific to *S. tuberosum*; this set is a valuable resource for other species within the *Solanum* genus.

The challenges of combining desirable alleles in heterozygous tetraploid genomes as potato can be diminished through a deeper understanding of its diversity and genome complexity. The haplotype-resolved genome assembly and protein coding gene prediction of DC, a variety cultivated in the Andean region of Colombia and Ecuador, contributes to this aim. The DC genome increases the repertoire of the recent high-quality haplotype-resolved assemblies of tetraploids (Bao *et al.* 2022; Hoopes *et al.* 2022; Sun *et al.* 2022). The DC study reported here, shows a high-quality genome assembly and annotation compared to other available genomes.

Further analysis of additional phased tetraploid potato genomes with highly divergent haplotypes i.e. in a pangenome, is considered a key contribution to design hybrids as new breeding schemes based on potato genomes (Zhang C *et al.* 2021; Hoopes *et al.* 2022). Moreover, the analyses of NLR gene clusters on haplotypes is desirable to combine resistance genes from specific haplotypes as part of these breeding schemes. The compiled pangenomes encompass a substantial gene pool derived from 44 diploid potatoes (Tang *et al.* 2022), six tetraploids from North America and Europe (Hoopes *et al.* 2022), and 296 diploids and polyploids from 60 species (Bozan *et al.* 2023). They collectively enhance the allelic diversity and gene repertoire of potato pangenomes. However, despite the wide distribution of the *S. tuberosum Andigenum* Group, spanning from northern Colombia to central Bolivia (Spooner *et al.* 2010), representation of tetraploid Andigenum Group accessions originating in the Andean region of Colombia is limited (see Supplementary Table S5). The DC assembly thus serves as the first representation of tetraploid Andigenum Group accessions originating from the Andean region of Colombia.

### Conclusion

In this study, a de novo assembly of a tetraploid Andigena variety namely DC (Diacol Capiro), was conducted by combining PacBio high-fidelity (HiFi) and proximity by ligation (HiC) sequencing. Illumina pair-end sequencing was used for technical validation. Hybrid assembly strategies resulted into a four haplotype-resolved genome assembly. This final DC assembly obtained is a reference-quality genome comparable to other high-quality genomes. Thus, the monoploid DMv6.1 and the diploid RH89-039-16 that correspond to haplotype resolved chromosome-scale genomes, have similar assembly metrics as compared to DC. The DC gene annotation also showed comparable BUSCO metrics to other high-quality tetraploid, diploid and monoploid genomes. Though DC showed differences in NLR content and domain combination with a reduced representation of TIR containing domains. Whether this might be related to its disease susceptibility to late blight and other pathogens awaits further investigation. DC is the first deep genome sequence of high assembly and annotation quality from a variety within the Andigenum group cultivated in the Andean region of Colombia and Ecuador, where centers of cultivated potato diversity arise. This assembled genome functions as a valuable instrument for improving the potato pangenome and formulating innovative breeding strategies aimed at developing superior, resilient, and more productive potato varieties. This contributes to a more profound comprehension of genome diversity and evolution in this prominent crop.

## Supplementary Material

jkae139_Supplementary_Data

## Data Availability

Raw data are available at the Bioproject PRJNA770354, Biosample SAMN22215911 (SRA accession SRR16302871) at NCBI. The genome assembly is also available in the NCBI under the accession number JAJHQB000000000. In addition, the custom pipelines and scripts for genome assembly, correction, and scaffolding used in this project are hosted on GitHub. This paper is also available in Spanish to facilitate discussions with Spanish-speaking audiences at https://github.com/phrh/DiacolCapiro_Genome. Supplemental material available at G3 online.
