# Supplementary material for: Chromosome-scale genome assembly and annotation of the tetraploid potato cultivar Diacol Capiro adapted to the Andean region
Source: G3 (Bethesda). 2024 Jul 26;14(9):jkae139. doi: 10.1093/g3journal/jkae139 (PMC11537804; doi:10.1093/g3journal/jkae139)
Supplement: jkae139_Supplementary_Data [file jkae139_supplementary_data.pdf]

Supplementary Figures and Tables: Chromosome-scale genome assembly and annotation of the tetraploid potato cultivar Diacol Capiro adapted to the Andean region

Paula H. Reyes-Herrera[1,]* *Diego A. Delgadillo-Duran[1]*
Mirella Flores-Gonzalez[2] *Lukas A. Mueller[2]* *Marco A. Cristancho[3]*
Luz Stella Barrero[1,]*

1. Corporación Colombiana de Investigación Agropecuaria (AGROSAVIA), Bogotá, Colombia
2. Boyce Thompson Institute, Ithaca, NY 14850, USA
3. Vicerrectoría de Investigación y Creación, Universidad de los Andes, Bogotá, Colombia,
∗ Corresponding authors: phreyes@agrosavia.co, lbarrero@agrosavia.co

List of Figures

S1 GenomeScope with HiFi reads. 3
S2 GenomeScope with Illumina reads. 4
S3 SmudgePlot with HiFi reads. 5
S4 Dotplot for 48 largest scaffolds from Strategy 2 (st2) vs DMv6.1. 6
S5 Dotplot for 48 largest scaffolds from Strategy 3 (st3) vs DMv6.1. 7
S6 Dotplot for 48 largest scaffolds from Strategy 6 (st6) vs DMv6.1. 8
S7 BUSCO score embriophyta_odb lineage for the chromosome 1 for hv2 (hybrid), st2, st6 and DMv6 for comparison. st3 is not present in the figure because it has the lowest median alignment rate to the DMv6 chr1. The chr1_hv2 was selected. 10
S8 BUSCO score embriophyta_odb lineage for the chromosome 2 for hv2 (hybrid), st2, st6, DMv6 and RH for comparison. st3 is not present in the figure because it has the lowest median alignment rate to the DMv6 chr1. For this selection the dotplot was also taken into account and the st2 was selected. 11
S9 BUSCO score embriophyta_odb lineage for the chromosome 3 for h2, h3 (hybrids), st2, st3, st6, DMv6 for comparison. The h3 was selected. 12
S10 BUSCO score embriophyta_odb lineage for the chromosome 4 for h2, h3 (hybrids), st2, st3, st6, DMv6 for comparison. The h2 was selected. 13
S11 BUSCO score embriophyta_odb lineage for the chromosome 5 for h2 (hybrid), st2, st3, st6, DMv6 for comparison. Since the st3 recovers only three scaffolds, the st2 was selected. 14
S12 BUSCO score embriophyta_odb lineage for the chromosome 6 for h2 (hybrid), st2, st3, st6, DMv6 for comparison. The st2 was selected. 15
S13 BUSCO score embriophyta_odb lineage for the chromosome 7 for h2 (hybrid), st2, st3, st6, DMv6 for comparison. The st2 was selected. 16
S14 BUSCO score embriophyta_odb lineage for the chromosome 8 for st2, st3, st6, DMv6 for comparison. The st2 was selected. In this case, the scaffolds aligned partially among them, for this reason there is not a hybrid chromosome. 17
S15 BUSCO score embriophyta_odb lineage for the chromosome 9 for h2 (hybrid) st2, st3, st6, DMv6 for comparison. The st3 was selected because the st6 recovers only three scaffolds . 18
S16 BUSCO score embriophyta_odb lineage for the chromosome 10 for st2, st3, st6, DMv6 for comparison. The st2 was selected, it was the only assembly that had four scaffolds for this chromosome. 19
S17 BUSCO score embriophyta_odb lineage for the chromosome 11 for h2, h3 (hybrids), st2, st3, st6 and DMv6. The st6 was selected. 20

S18 BUSCO score embriophyta_odb lineage for the chromosome 12 for h2 (hybrid), st2, st3, st6 and DMv6. The st6 was selected. 21
S19 Boxplot with the median alignment rate between each strategy st2, st3 and st6 and the DMv6.1 for each chromosome. 22
S20 Heatmap for the Hi-C filtered data alignment to the DC Assembly. 24
S21 Repeat content composition found in the DC Assembly. 25
S22 Barplot illustrating gene predictions distributed across individual chromosomes. Each bar represents the count of gene predictions for a specific chromosome within the genome assembly. 29

List of Tables

S1 Comparison for the eight assemblies. Here, we present metrics at a chromosome-scale such as median, standard deviation between the four largest scaffolds, and BUSCO score for all the chromosomes. In color the standard deviation for the selected assemblies (st2, st3 and st6) that are the three lowest ones. 9
S2 Pseudo-chromosome selection for each homologous group. The selected scaffolds for the DC assembly are highlighted in color. 23
S3 Metadata for the datasets used for the DC assembly annotation. 26
S4 Comparative Genome Assembly Statistics for Solanum tuberosum Chromosome-Scale Assemblies 28
S5 Solanum tuberosum group Andigenum (Andigena) genomes (Bozan et al., 2023) and the origin of the material sourced from Genesys (https://www.genesys-pgr.org/). The genome sequencing data for most of these accessions comes from the Illumina platform, except for ADG1 from Peru (blue background).) 31

1 Size, ploidy, and heterozygosity estimation

1.1 GenomeScope Illumina and Hifi

1.1.1 HiFi reads

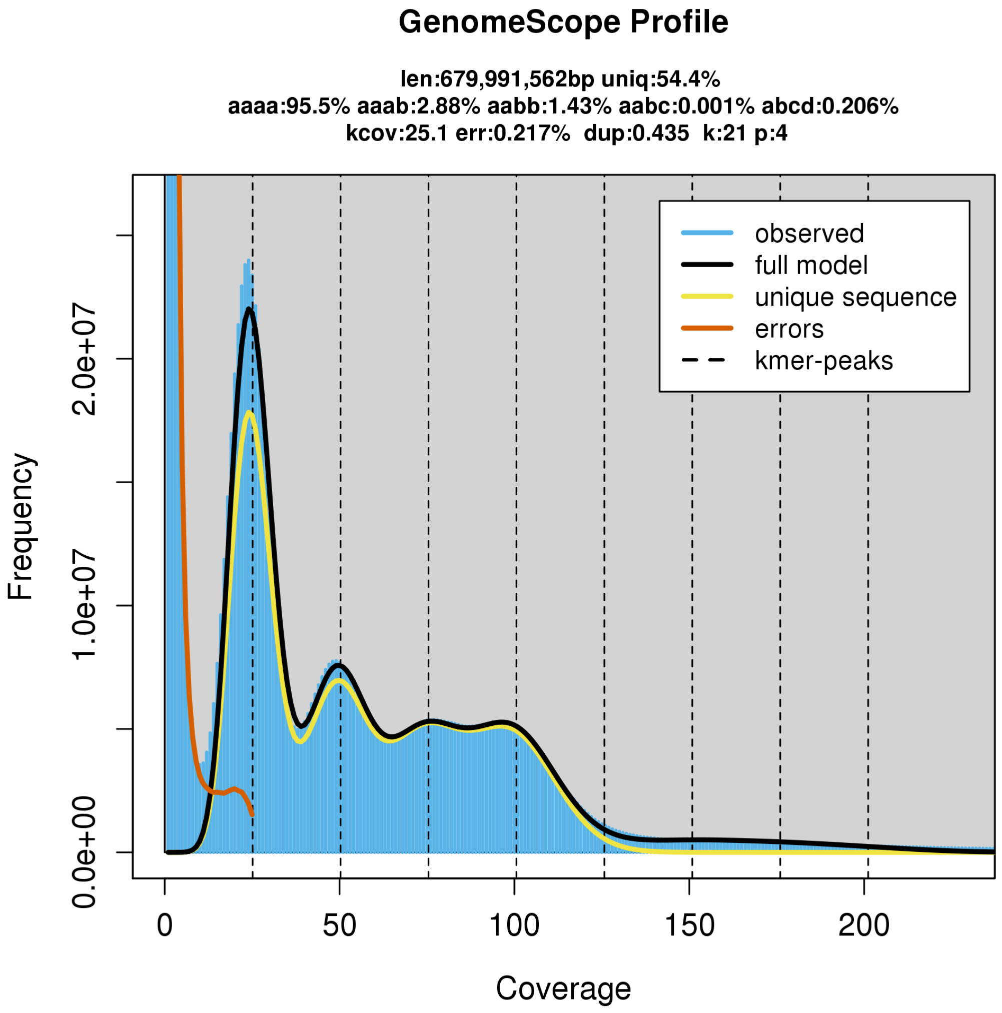

Fig. S1: GenomeScope with HiFi reads.

1.1.2 Illumina reads

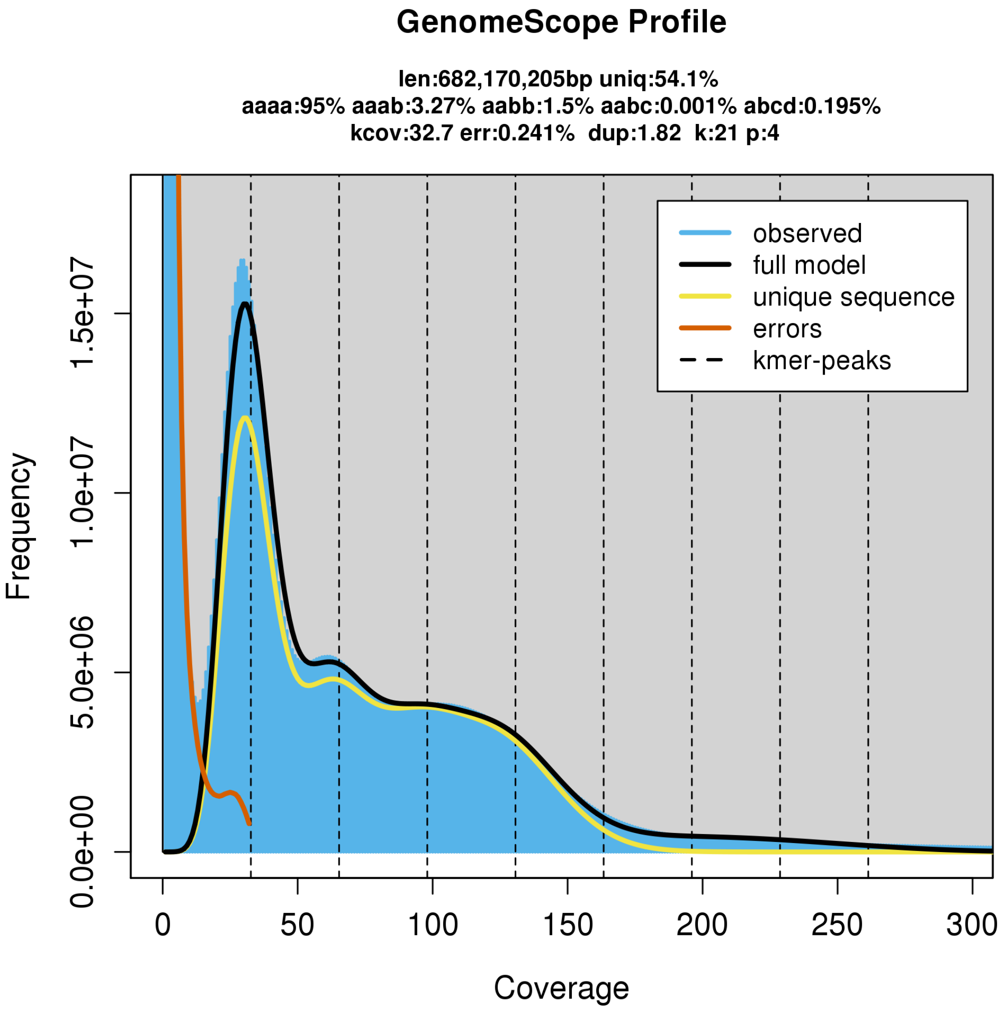

Fig. S2: GenomeScope with Illumina reads.

1.2 Smudgeplot ploidy

1.2.1 HiFi reads

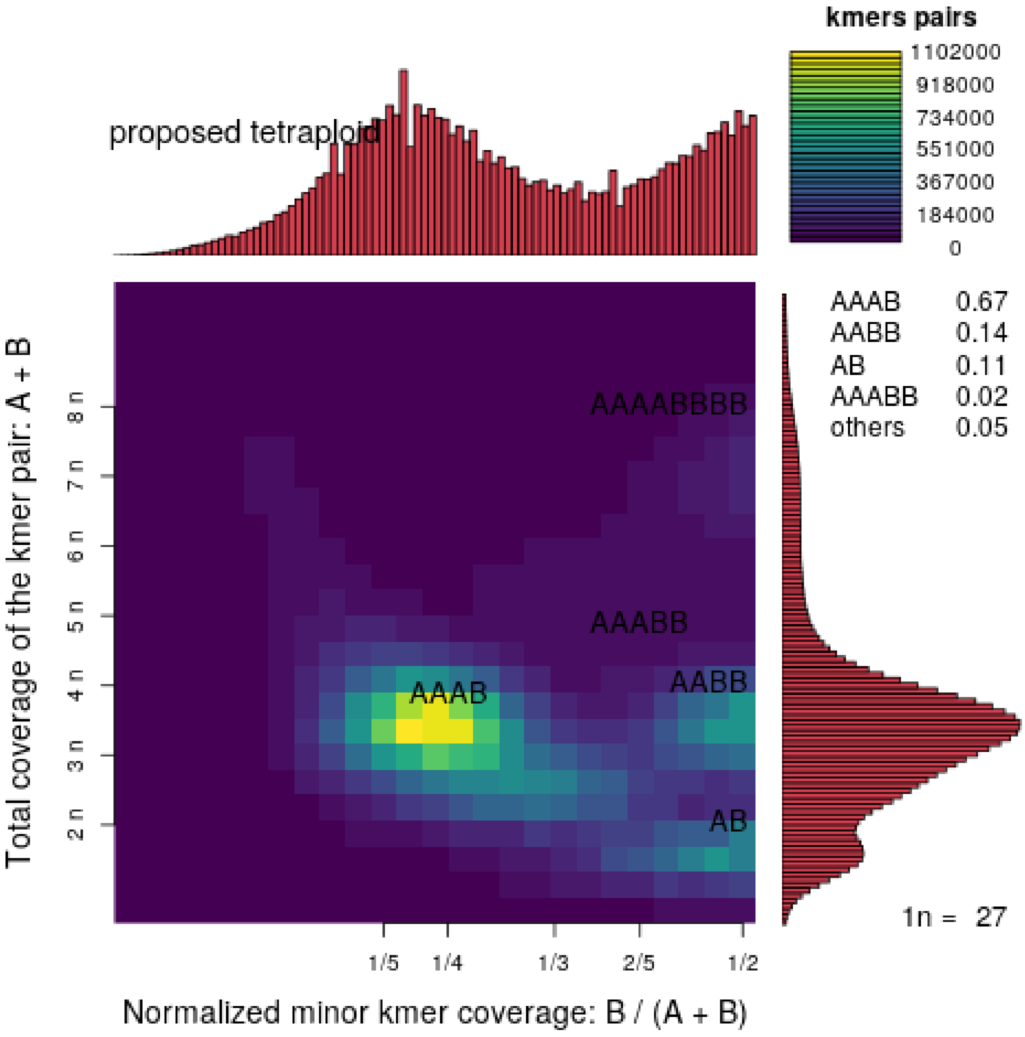

Fig. S3: SmudgePlot with HiFi reads.

2 Dotplot using as reference DMv6.1

2.1 st2

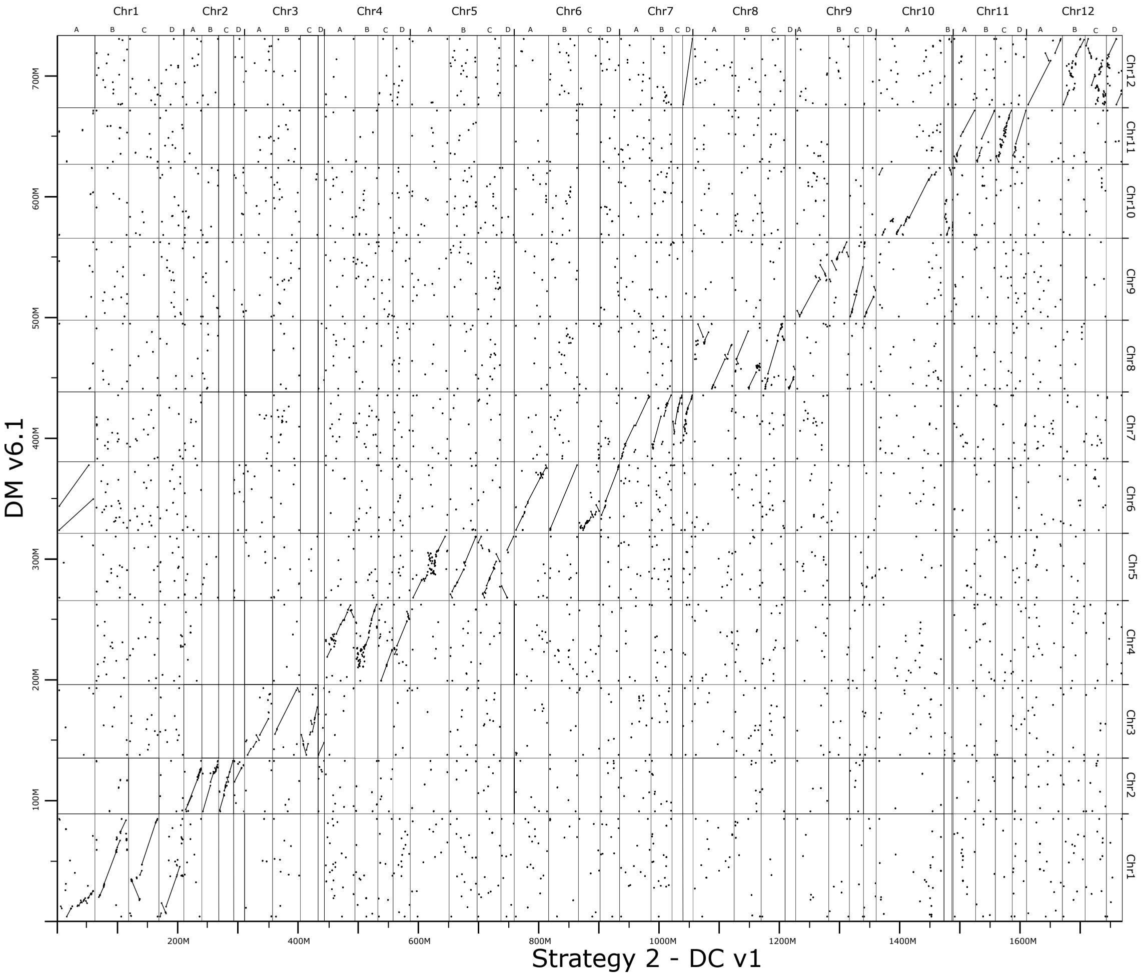

Fig. S4: Dotplot for 48 largest scaffolds from Strategy 2 (st2) vs DMv6.1.

2.2 st3

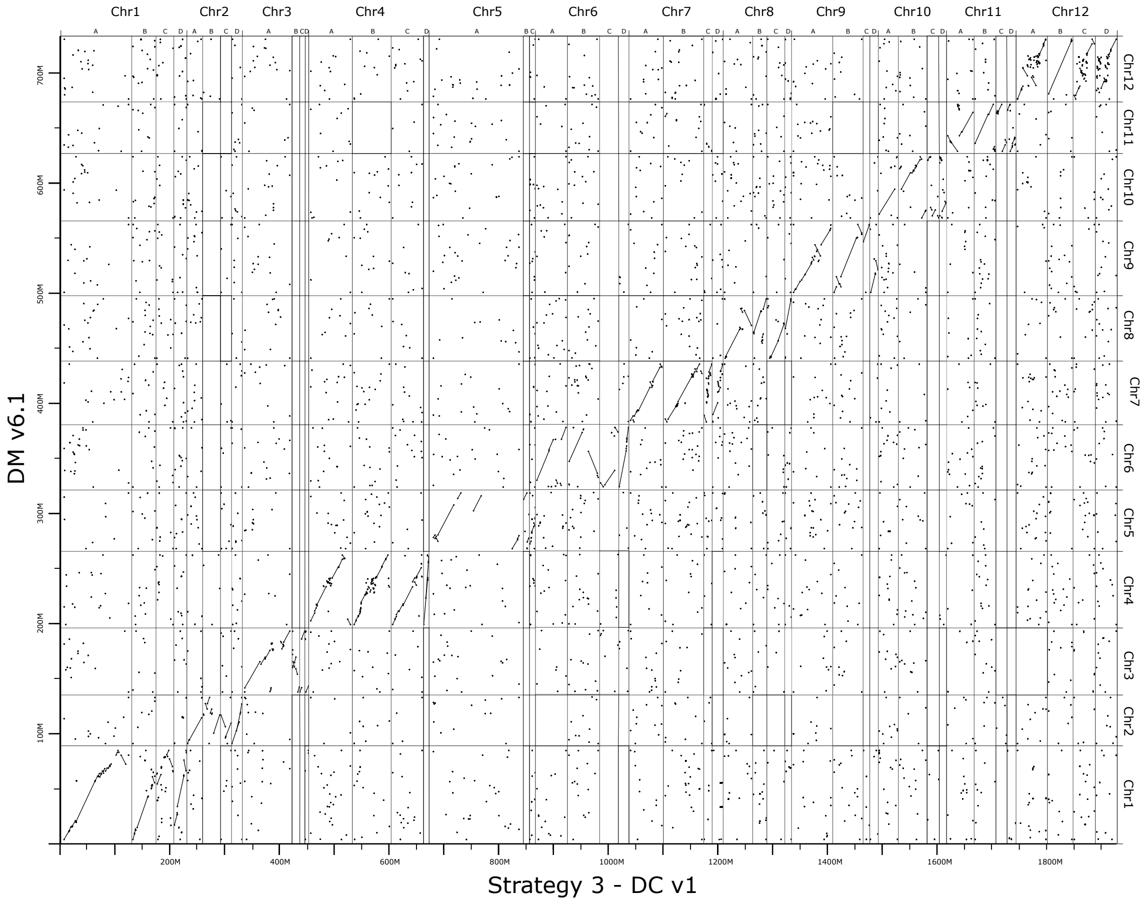

Fig. S5: Dotplot for 48 largest scaffolds from Strategy 3 (st3) vs DMv6.1.

2.3 st6

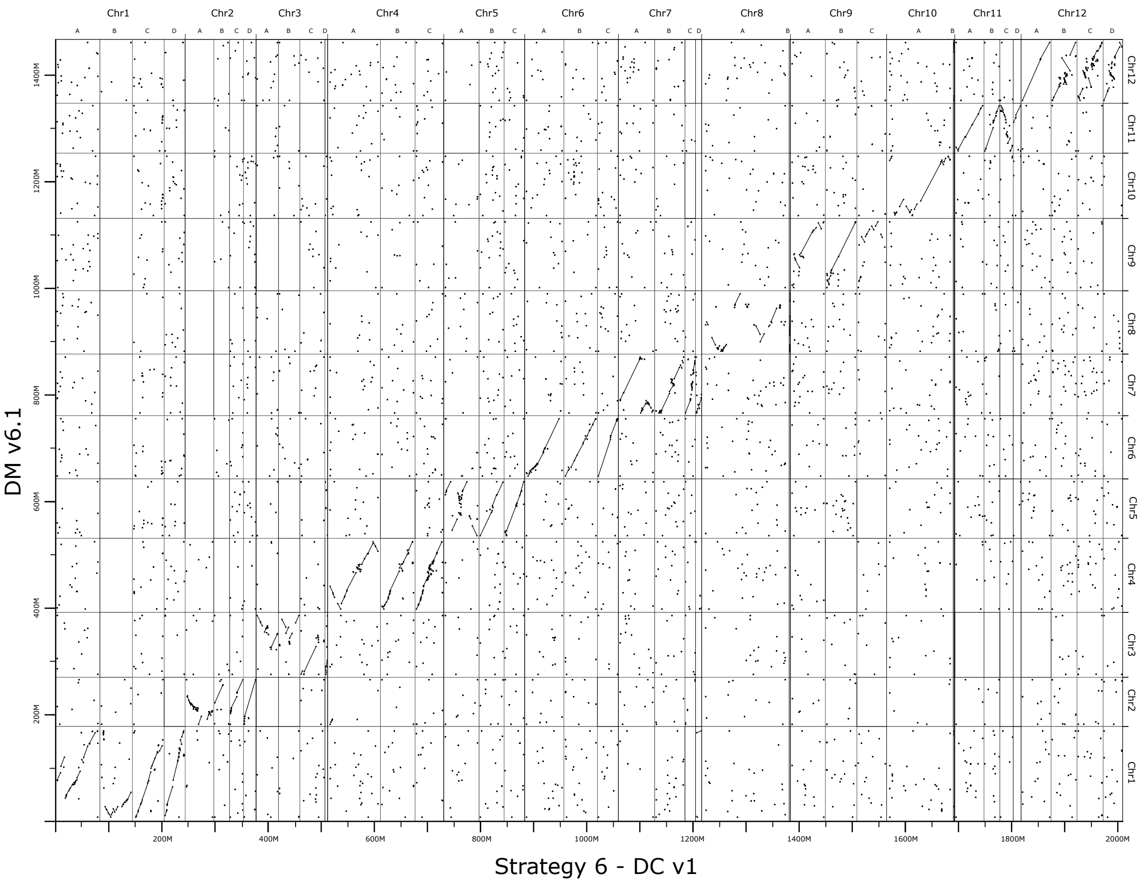

Fig. S6: Dotplot for 48 largest scaffolds from Strategy 6 (st6) vs DMv6.1.

3 AllHiC assemblies comparison

Chromosome	Metric	St0	St1	St2	St3	St4	St5	St6	St7	DC Assembly
Chr1	Median	21,741,502	3,110,768	53,285,777	38,628,058	2,160,440	5,079,489	60,432,614	553,750	59,148,421
	Standard dev.	109,869,321	115,008,439	8,786,433	48,024,492	115,552,867	137,215,411	18,096,930	103,285,001	14,685,453
Chr2	Median	26,711,798	24,372,284	26,756,512	24,853,756	19,107,354	18,069,345	28,374,506	451,850	26,756,512
	Standard dev.	21,019,704	41,984,567	5,072,662	6,264,057	46,488,337	41,756,556	13,736,625	60,930,504	5,072,662
Chr3	Median	16,989,331	2,466,086	38,085,496	12,311,639	1,905,349	8,771,432	41,417,524	1,317,550	44,382,623
	Standard dev.	38,852,782	61,897,225	16,920,785	39,791,770	61,810,038	58,236,523	19,033,805	64,269,172	7,954,821
Chr4	Median	32,015,992	1,987,441	33,596,155	64,828,588	901,720	712,357	59,667,761	2,441,807	59,667,761
	Standard dev.	38,903,873	94,527,201	11,301,982	30,625,402	96,374,452	120,159,752	40,943,792	86,638,638	25,613,914
Chr5	Median	1,131,241	35,669,682	43,266,428	11,720,428	20,013,454	54,161,302	43,090,596	45,150,869	43,266,428
	Standard dev.	95,425,685	44,856,591	17,336,879	81,388,959	55,875,142	31,887,064	27,672,889	23,697,526	17,336,879
Chr6	Median	34,998,886	33,696,198	42,980,718	46,896,032	35,781,931	94,316,715	51,461,440	13,884,160	42,980,718
	Standard dev.	31,313,054	46,141,574	11,419,470	19,340,291	37,679,345	132,115,573	32,502,619	58,152,336	11,419,470
Chr7	Median	32,401,394	41,810,444	26,701,425	41,718,899	35,525,584	89,750,149	38,765,554	39,536,505	41,651,191
	Standard dev.	32,180,521	10,119,146	16,529,481	29,159,335	12,060,581	123,558,620	27,322,169	30,046,939	28,537,448
Chr8	Median	25,426,249	2,573,850	42,542,851	29,658,846	2,958,903	11,238,566	1,916,924	1,455,616	42,542,851
	Standard dev.	25,594,326	72,569,169	20,626,865	17,174,316	71,267,827	74,313,939	81,045,295	65,164,907	20,626,865
Chr9	Median	33,545,031	31,921,259	29,300,676	35,707,381	27,247,351	6,527,319	57,694,088	472,959	35,707,381
	Standard dev.	27,130,679	13,140,248	15,226,355	30,392,086	8,380,691	84,373,177	30,109,522	81,342,090	30,392,086
Chr10	Median	10,680,393	3,420,772	8,302,247	29,844,266	3,894,350	5,604,687	2,206,853	2,311,361	29,844,266
	Standard dev.	46,722,258	56,462,186	53,393,289	16,927,167	57,625,031	55,263,532	61,466,334	54,781,830	16,927,167
Chr11	Median	30,470,915	39,712,154	30,832,629	30,372,349	23,567,552	70,732,312	27,905,633	30,585,454	27,905,633
	Standard dev.	16,994,497	21,426,206	5,772,522	15,934,515	19,755,445	97,973,342	16,821,382	13,376,078	16,821,382
Chr12	Median	21,598,563	34,306,738	36,868,061	43,923,677	33,755,394	57,800,693	49,134,161	42,685,390	49,134,161
	Standard dev.	84,806,723	8,888,636	13,911,044	7,945,528	10,646,269	40,821,771	7,995,958	3,855,400	7,995,958
All Chromosomes	Median	25,182,743	25,664,826	35,538,570	32,977,280	22,676,497	7,136,060	41,417,524	3,581,318	43,707,338
	Standard dev.	50,533,883	51,869,796	19,227,179	32,037,745	52,606,859	74,286,606	33,865,942	53,970,752	19,784,538
	% Complete BUSCO Embryophyta	99.4	99.2	99.3	99.3	99.3	99.5	99.6	99.3	99.4

Tab. S1: Comparison for the eight assemblies. Here, we present metrics at a chromosome-scale such as median, standard deviation between the four largest scaffolds, and BUSCO score for all the chromosomes. In color the standard deviation for the selected assemblies (st2, st3 and st6) that are the three lowest ones.

4 Pseudo-chromosome selection

4.1 BUSCO scores

4.1.1 Chromosome 1

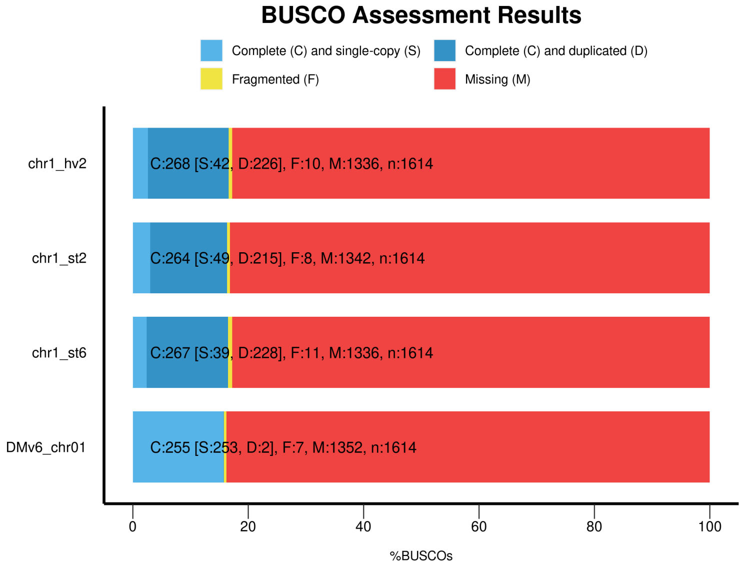

Fig. S7: BUSCO score embriophyta_odb lineage for the chromosome 1 for hv2 (hybrid), st2, st6 and DMv6 for comparison. st3 is not present in the figure because it has the lowest median alignment rate to the DMv6 chr1. The chr1_hv2 was selected.

4.1.2 Chromosome 2

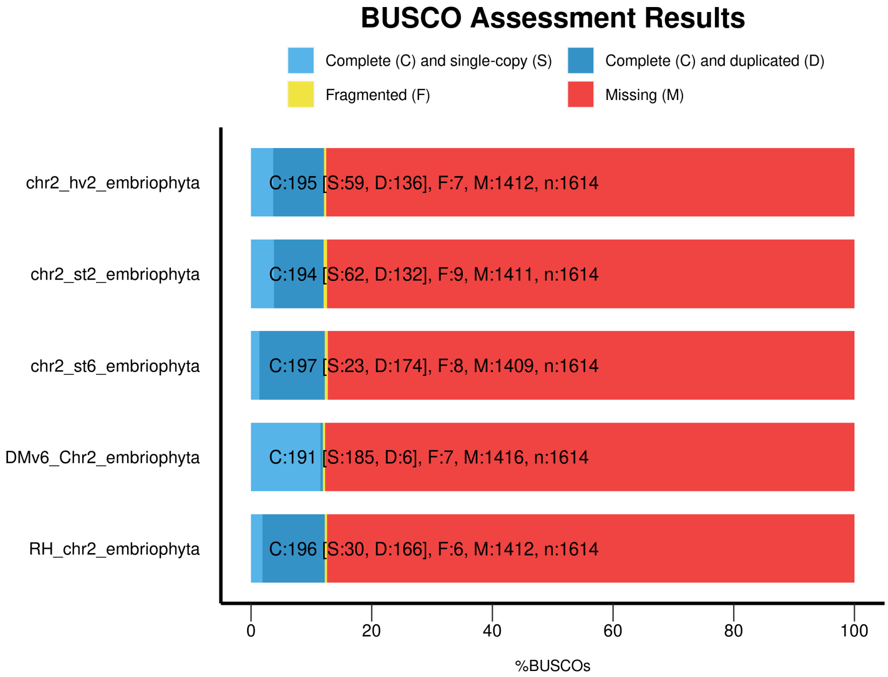

Fig. S8: BUSCO score embriophyta_odb lineage for the chromosome 2 for hv2 (hybrid), st2, st6, DMv6 and RH for comparison. st3 is not present in the figure because it has the lowest median alignment rate to the DMv6 chr1. For this selection the dotplot was also taken into account and the st2 was selected.

4.1.3 Chromosome 3

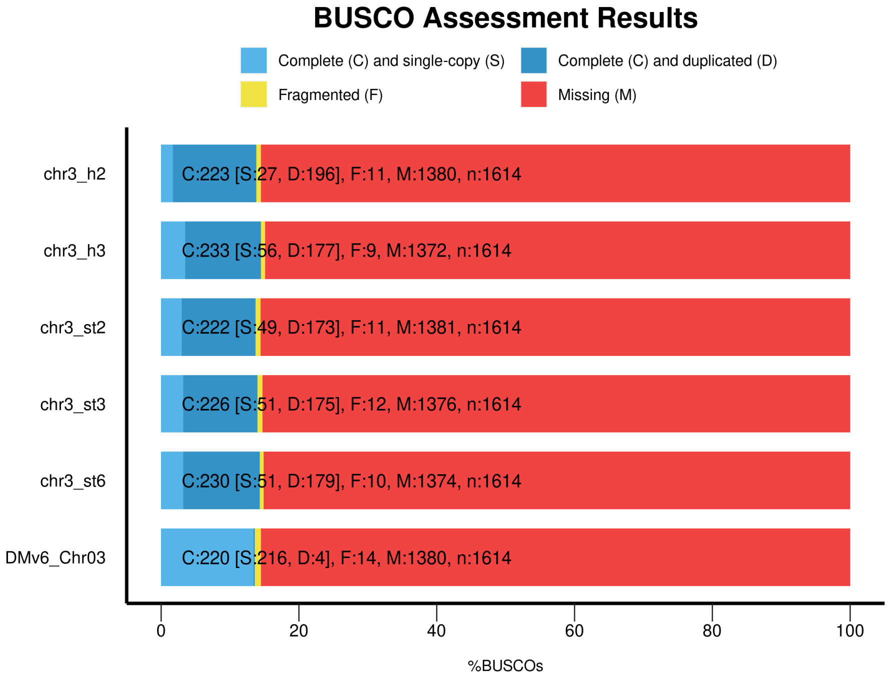

Fig. S9: BUSCO score embriophyta_odb lineage for the chromosome 3 for h2, h3 (hybrids), st2, st3, st6, DMv6 for comparison. The h3 was selected.

4.1.4 Chromosome 4

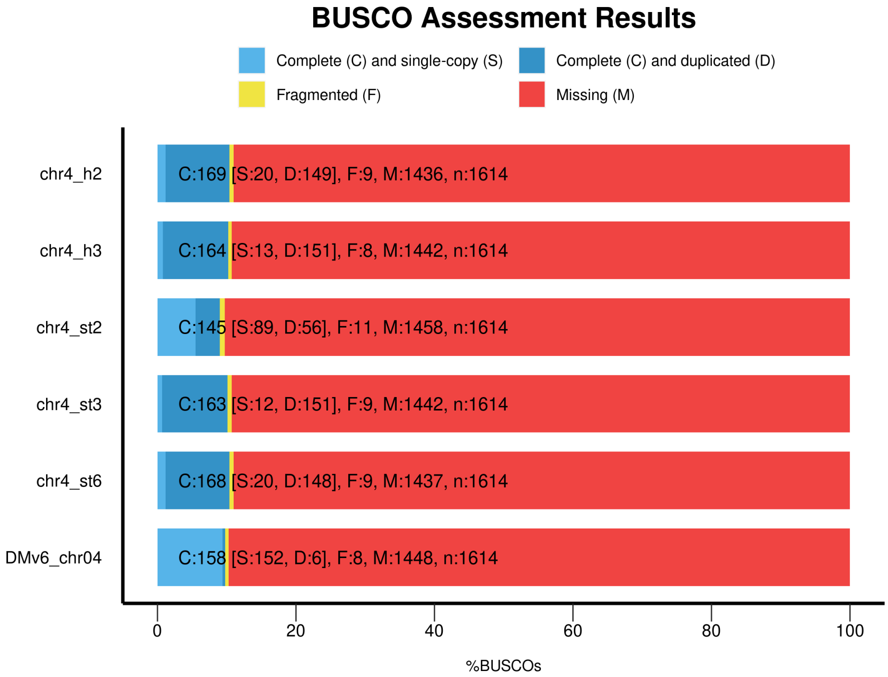

Fig. S10: BUSCO score embriophyta_odb lineage for the chromosome 4 for h2, h3 (hybrids), st2, st3, st6, DMv6 for comparison. The h2 was selected.

4.1.5 Chromosome 5

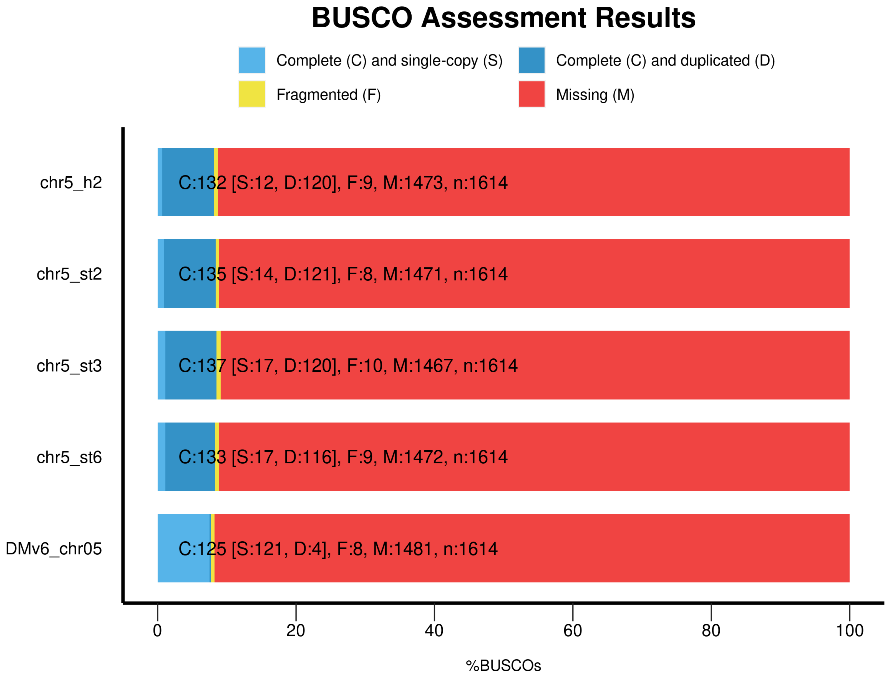

Fig. S11: BUSCO score embriophyta_odb lineage for the chromosome 5 for h2 (hybrid), st2, st3, st6, DMv6 for comparison. Since the st3 recovers only three scaffolds, the st2 was selected.

4.1.6 Chromosome 6

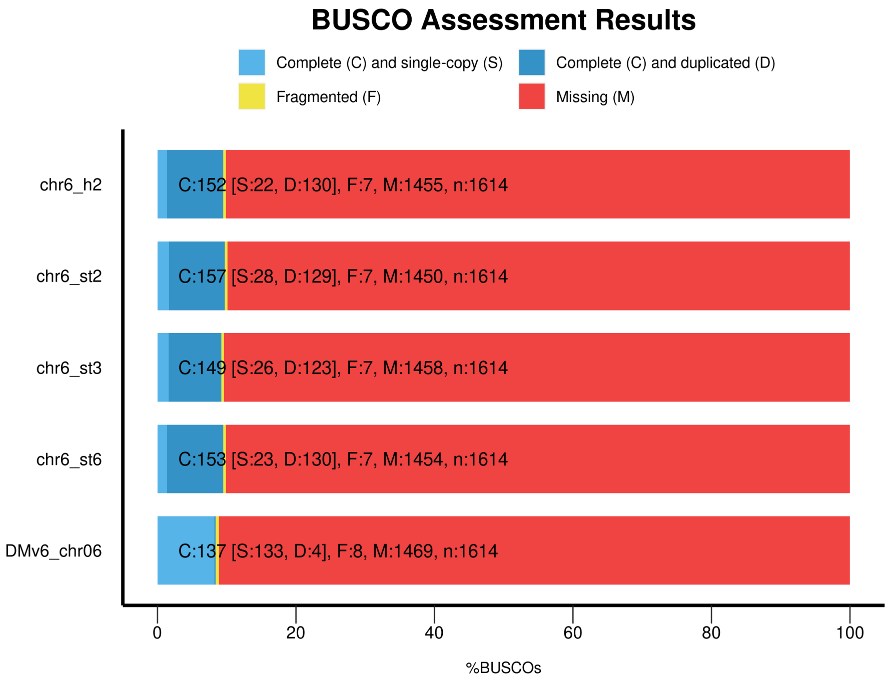

Fig. S12: BUSCO score embriophyta_odb lineage for the chromosome 6 for h2 (hybrid), st2, st3, st6, DMv6 for comparison. The st2 was selected.

4.1.7 Chromosome 7

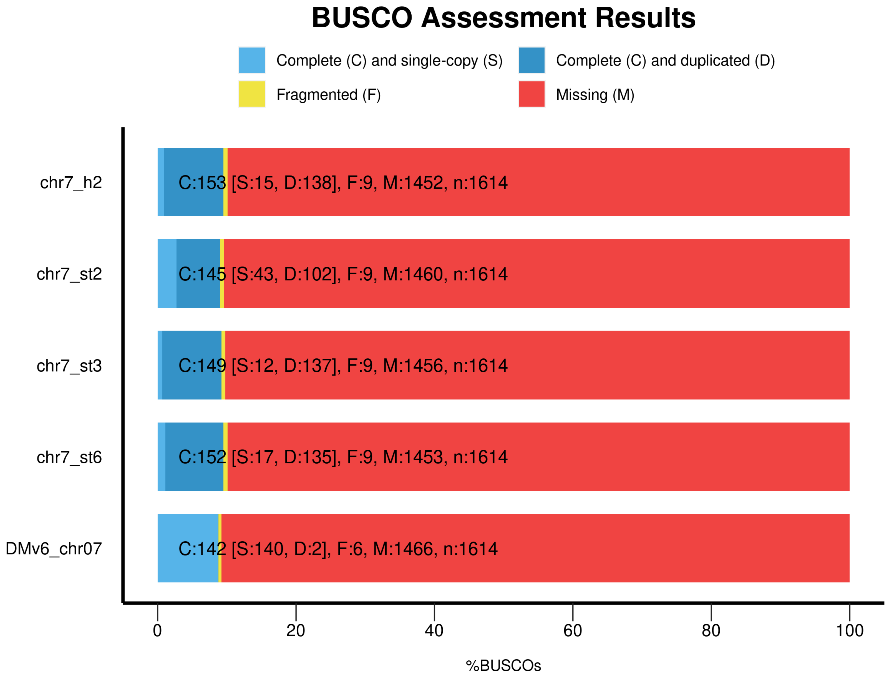

Fig. S13: BUSCO score embriophyta_odb lineage for the chromosome 7 for h2 (hybrid), st2, st3, st6, DMv6 for comparison. The st2 was selected.

4.1.8 Chromosome 8

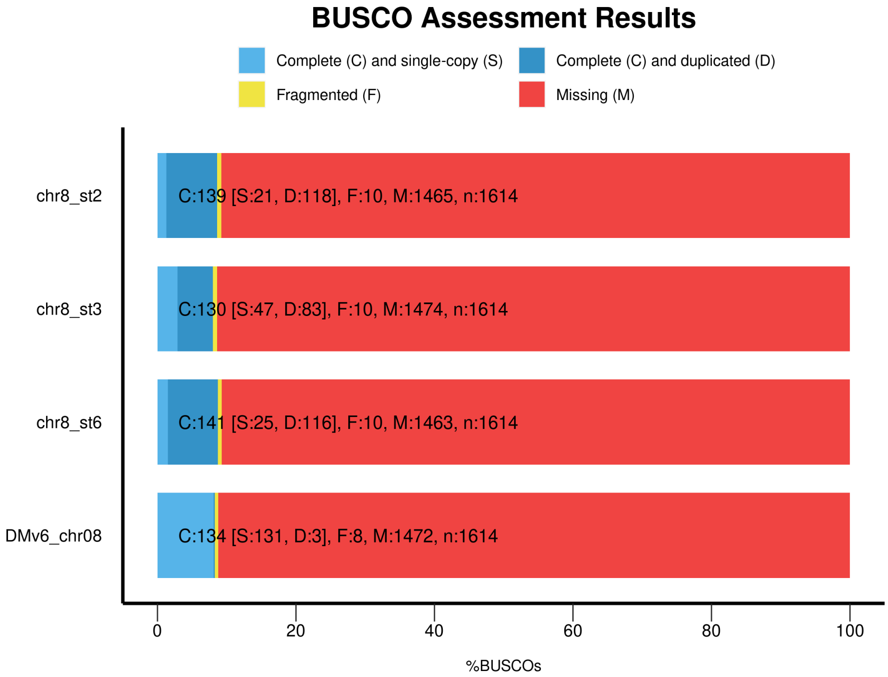

Fig. S14: BUSCO score embriophyta_odb lineage for the chromosome 8 for st2, st3, st6, DMv6 for comparison. The st2 was selected. In this case, the scaffolds aligned partially among them, for this reason there is not a hybrid chromosome.

4.1.9 Chromosome 9

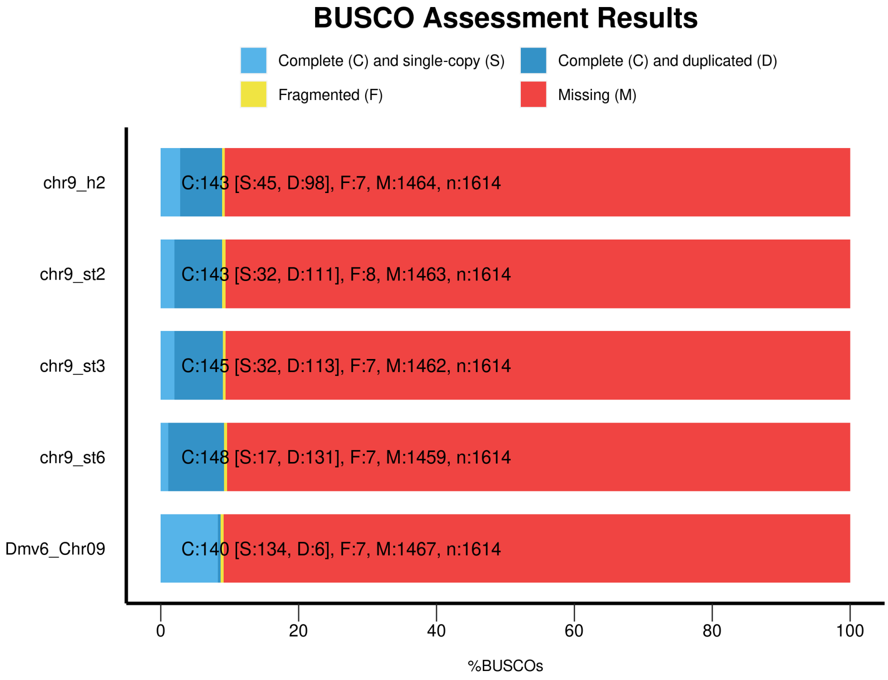

Fig. S15: BUSCO score embriophyta_odb lineage for the chromosome 9 for h2 (hybrid) st2, st3, st6, DMv6 for comparison. The st3 was selected because the st6 recovers only three scaffolds

4.1.10 Chromosome 10

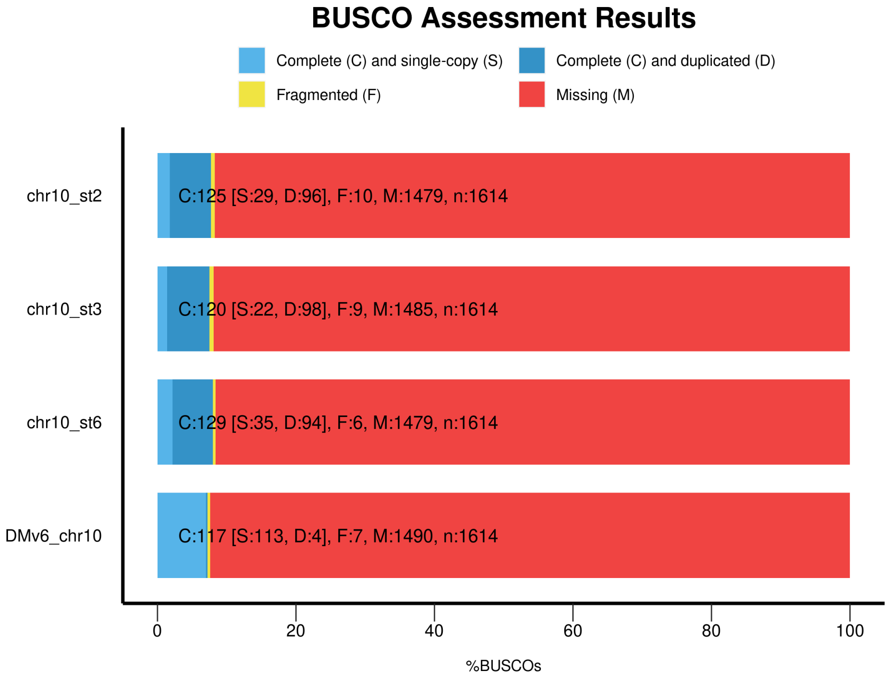

Fig. S16: BUSCO score embriophyta_odb lineage for the chromosome 10 for st2, st3, st6, DMv6 for comparison. The st2 was selected, it was the only assembly that had four scaffolds for this chromosome.

4.1.11 Chromosome 11

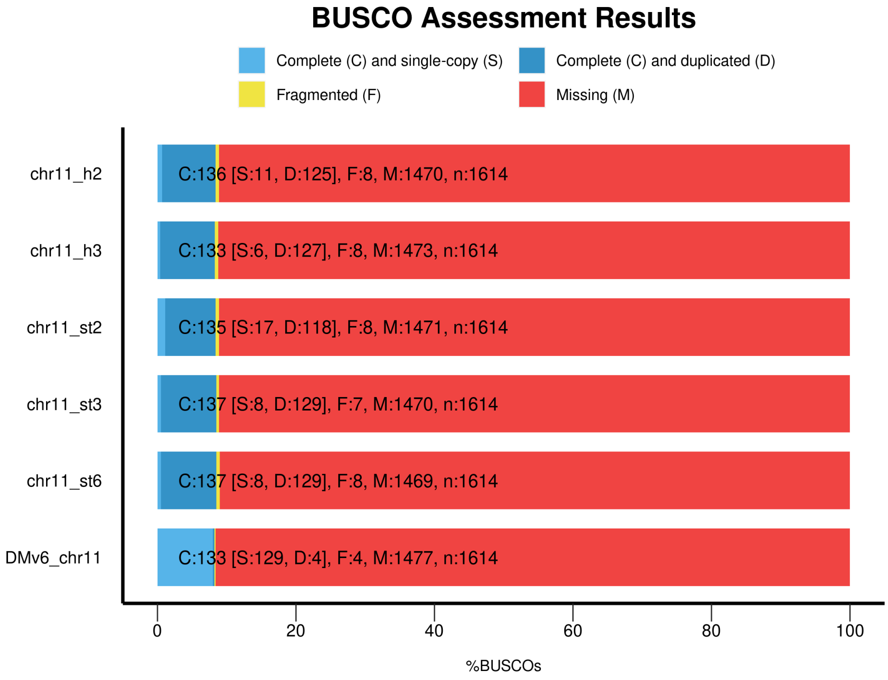

Fig. S17: BUSCO score embriophyta_odb lineage for the chromosome 11 for h2, h3 (hybrids), st2, st3, st6 and DMv6. The st6 was selected.

4.1.12 Chromosome 12

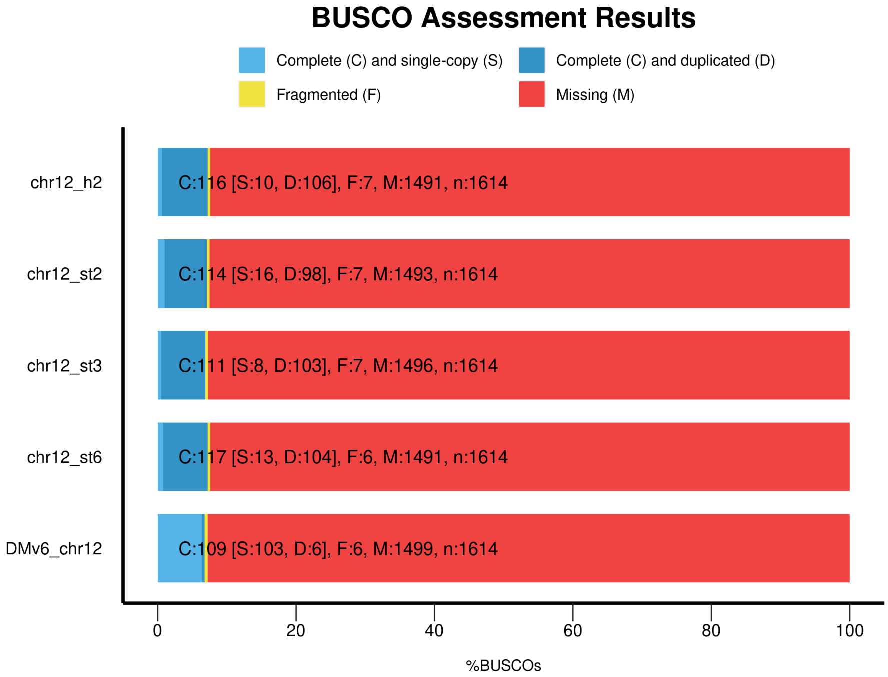

Fig. S18: BUSCO score embriophyta_odb lineage for the chromosome 12 for h2 (hybrid), st2, st3, st6 and DMv6. The st6 was selected.

5 Median alignment rate

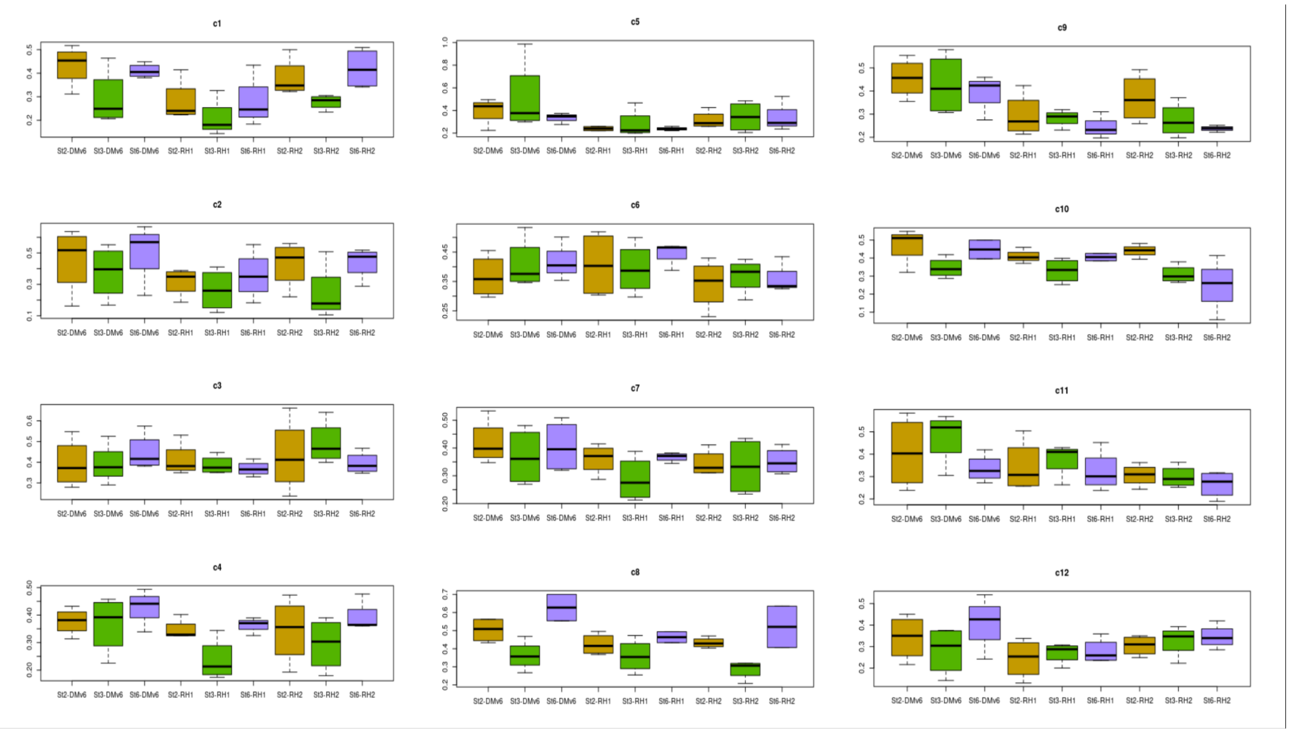

Fig. S19: Boxplot with the median alignment rate between each strategy st2, st3 and st6 and the DMv6.1 for each chromosome.

5.1 Chromosome-scale results

Chromosome	Scaffold	st2	st3	st6
Chr1	group1	62.315.048	128.484.464	84.058.789
	group2	55.981.793	44.369.351	61.138.456
	group3	50.589.760	32.886.765	59.726.771
	group4	41.566.182	24.303.063	39.803.751
Chr2	group1	30.400.441	29.129.414	54.035.364
	group2	28.239.167	32.858.066	30.073.183
	group3	25.273.857	20.578.097	26.675.828
	group4	18.737.376	20.362.709	24.209.259
Chr3	group1	46.713.341	90.158.580	42.350.143
	group2	46.415.103	14.497.564	40.484.904
	group3	29.755.889	10.125.713	47.419.620
	group4	11.060.547	7.699.189	5.804.379
Chr4	group1	38.325.480	79.274.433	98.785.543
	group2	25.780.134	70.587.683	65.213.751
	group3	28.866.830	59.069.493	54.121.771
	group4	25.315.846	10.671.675	157.89
Chr5	group1	64.452.807	170.413.983	66.476.228
	group2	46.806.292	11.938.381	47.203.898
	group3	39.726.563	11.502.474	38.977.294
	group4	22.582.105	500.713	567.672
Chr6	group1	57.137.420	58.426.413	73.567.176
	group2	49.686.225	58.682.480	63.120.951
	group3	36.275.211	35.365.650	39.801.929
	group4	32.747.381	18.916.069	274.1
Chr7	group1	52.274.013	62.839.024	68.103.394
	group2	35.377.714	73.326.350	57.067.751
	group3	18.025.136	15.874.127	20.463.357
	group4	17.594.447	20.598.773	12.107.563
Chr8	group1	68.391.044	54.024.279	163.405.974
	group2	45.064.533	26.249.896	3.028.748
	group3	40.021.169	33.067.795	805.1
	group4	18.128.314	12.858.201	167.972
Chr9	group1	54.939.629	74.963.870	65.613.138
	group2	34.681.966	55.369.174	59.274.238
	group3	23.919.386	12.820.977	56.113.937
	group4	21.542.729	16.045.588	635.098
Chr10	group1	112.030.686	36.972.116	124.455.767
	group2	13.824.493	52.432.724	2.446.506
	group3	2.780.000	22.716.415	1.967.199
	group4	950.199	13.771.054	201.099
Chr11	group1	37.474.559	50.491.741	55.027.943
	group2	33.229.245	40.109.622	30.300.319
	group3	28.436.012	20.635.076	25.510.947
	group4	24.172.047	17.004.373	15.436.885
Chr12	group1	59.583.815	57.332.846	56.432.154
	group2	38.036.695	46.826.656	49.017.343
	group3	35.699.426	41.020.697	49.250.978
	group4	26.783.390	40.013.336	37.132.070

Tab. S2: Pseudo-chromosome selection for each homologous group. The selected scaffolds for the DC assembly are highlighted in color.

6 DC Assembly HiC Heatmap

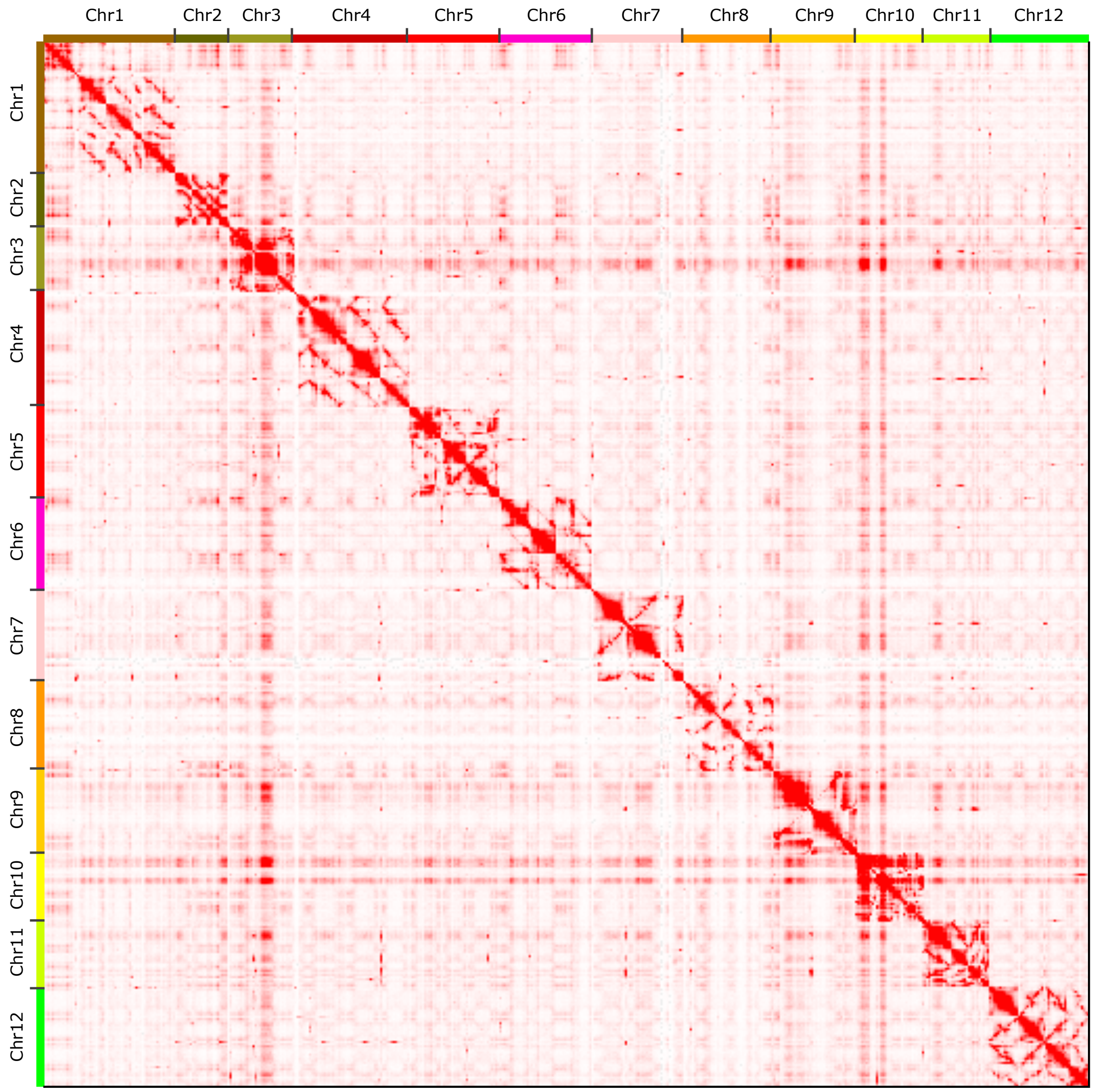

Fig. S20: Heatmap for the Hi-C filtered data alignment to the DC Assembly.

7 Repeat Content

```
==================================================
file name: DCH2-v3.fasta
sequences:          1194
total length: 2369620969 bp  (2368774669 bp excl N/X-runs)
GC level:         34.68 %
bases masked: 1037746294 bp ( 43.79 %)
==================================================
               number of      length   percentage
               elements*    occupied  of sequence
--------------------------------------------------
Retroelements       677744    866456440 bp   36.57 %
   SINEs:            48578      6379342 bp    0.27 %
   Penelope             34         2874 bp    0.00 %
   LINEs:            77432     38276696 bp    1.62 %
    CRE/SLACS            0            0 bp    0.00 %
     L2/CR1/Rex          0            0 bp    0.00 %
     R1/LOA/Jockey       0            0 bp    0.00 %
     R2/R4/NeSL          8          357 bp    0.00 %
     RTE/Bov-B       54832     13388045 bp    0.56 %
     L1/CIN4         30080     25818754 bp    1.09 %
   LTR elements:    551734    821800402 bp   34.68 %
     BEL/Pao             0            0 bp    0.00 %
     Ty1/Copia       89593     96051690 bp    4.05 %
     Gypsy/DIRS1    440786    693390905 bp   29.26 %
       Retroviral        0            0 bp    0.00 %

DNA transposons     356285    113247983 bp    4.78 %
   hobo-Activator    78259     25042998 bp    1.06 %
   Tc1-IS630-Pogo    82545     16457195 bp    0.69 %
   En-Spm                0            0 bp    0.00 %
   MuDR-IS905            0            0 bp    0.00 %
   PiggyBac              0            0 bp    0.00 %
   Tourist/Harbinger 47410     12544047 bp    0.53 %
   Other (Mirage,        8          422 bp    0.00 %
    P-element, Transib)

Rolling-circles          0            0 bp    0.00 %

Unclassified:        29206     11049983 bp    0.47 %

Total interspersed repeats:   990754406 bp   41.81 %


Small RNA:           25633      3969903 bp    0.17 %

Satellites:           9049     10195729 bp    0.43 %
Simple repeats:     593591     28605196 bp    1.21 %
Low complexity:     139120      8074775 bp    0.34 %
--------------------------------------------------
```

Fig. S21: Repeat content composition found in the DC Assembly.

8 RNAseq used for the annotation

Tab. S3: Metadata for the datasets used for the DC assembly annotation.

Public datasets							
Run	LibraryStrategy	LibraryLayout	Platform	Model	BioProject	ScientificName	SampleName
SRR21209942	RNA-Seq	SINGLE	ILLUMINA	Illumina HiSeq 2000	PRJNA874012	Solanum tuberosum subsp. andigenum	AA_TLC-1
SRR21209941	RNA-Seq	SINGLE	ILLUMINA	Illumina HiSeq 2000	PRJNA874012	Solanum tuberosum subsp. andigenum	AB_TRC-1
SRR21209930	RNA-Seq	SINGLE	ILLUMINA	Illumina HiSeq 2000	PRJNA874012	Solanum tuberosum subsp. andigenum	AC_SLC-1
SRR21209919	RNA-Seq	SINGLE	ILLUMINA	Illumina HiSeq 2000	PRJNA874012	Solanum tuberosum subsp. andigenum	AD_SRC-1
SRR21209908	RNA-Seq	SINGLE	ILLUMINA	Illumina HiSeq 2000	PRJNA874012	Solanum tuberosum subsp. andigenum	AE_TL3-1
SRR21209899	RNA-Seq	SINGLE	ILLUMINA	Illumina HiSeq 2000	PRJNA874012	Solanum tuberosum subsp. andigenum	AF_TR3-1
SRR21209898	RNA-Seq	SINGLE	ILLUMINA	Illumina HiSeq 2000	PRJNA874012	Solanum tuberosum subsp. andigenum	AG_SL3-1
SRR21209897	RNA-Seq	SINGLE	ILLUMINA	Illumina HiSeq 2000	PRJNA874012	Solanum tuberosum subsp. andigenum	AH_SR3-1
SRR21209896	RNA-Seq	SINGLE	ILLUMINA	Illumina HiSeq 2000	PRJNA874012	Solanum tuberosum subsp. andigenum	AI_TL1-1
SRR21209895	RNA-Seq	SINGLE	ILLUMINA	Illumina HiSeq 2000	PRJNA874012	Solanum tuberosum subsp. andigenum	AJ_TR1-1
SRR21209940	RNA-Seq	SINGLE	ILLUMINA	Illumina HiSeq 2000	PRJNA874012	Solanum tuberosum subsp. andigenum	AK_SL1-1
SRR21209939	RNA-Seq	SINGLE	ILLUMINA	Illumina HiSeq 2000	PRJNA874012	Solanum tuberosum subsp. andigenum	AL_SR1-1
SRR21209938	RNA-Seq	SINGLE	ILLUMINA	Illumina HiSeq 2000	PRJNA874012	Solanum tuberosum subsp. andigenum	AM_TL2-1
SRR21209937	RNA-Seq	SINGLE	ILLUMINA	Illumina HiSeq 2000	PRJNA874012	Solanum tuberosum subsp. andigenum	AN_TR2-1
SRR21209936	RNA-Seq	SINGLE	ILLUMINA	Illumina HiSeq 2000	PRJNA874012	Solanum tuberosum subsp. andigenum	AO_SL2-1
SRR21209935	RNA-Seq	SINGLE	ILLUMINA	Illumina HiSeq 2000	PRJNA874012	Solanum tuberosum subsp. andigenum	AP_SR2-1
SRR21209934	RNA-Seq	SINGLE	ILLUMINA	Illumina HiSeq 2000	PRJNA874012	Solanum tuberosum subsp. andigenum	BA_TLC-2
SRR21209933	RNA-Seq	SINGLE	ILLUMINA	Illumina HiSeq 2000	PRJNA874012	Solanum tuberosum subsp. andigenum	BB_TRC-2
SRR21209932	RNA-Seq	SINGLE	ILLUMINA	Illumina HiSeq 2000	PRJNA874012	Solanum tuberosum subsp. andigenum	BC_SLC-2
SRR21209931	RNA-Seq	SINGLE	ILLUMINA	Illumina HiSeq 2000	PRJNA874012	Solanum tuberosum subsp. andigenum	BD_SRC-2
SRR21209929	RNA-Seq	SINGLE	ILLUMINA	Illumina HiSeq 2000	PRJNA874012	Solanum tuberosum subsp. andigenum	BE_TL3-2
SRR21209928	RNA-Seq	SINGLE	ILLUMINA	Illumina HiSeq 2000	PRJNA874012	Solanum tuberosum subsp. andigenum	BF_TR3-2
SRR21209927	RNA-Seq	SINGLE	ILLUMINA	Illumina HiSeq 2000	PRJNA874012	Solanum tuberosum subsp. andigenum	BG_SL3-2
SRR21209926	RNA-Seq	SINGLE	ILLUMINA	Illumina HiSeq 2000	PRJNA874012	Solanum tuberosum subsp. andigenum	BH_SR3-2
SRR21209925	RNA-Seq	SINGLE	ILLUMINA	Illumina HiSeq 2000	PRJNA874012	Solanum tuberosum subsp. andigenum	BI_TL1-2
SRR21209924	RNA-Seq	SINGLE	ILLUMINA	Illumina HiSeq 2000	PRJNA874012	Solanum tuberosum subsp. andigenum	BJ_TR1-2
SRR21209923	RNA-Seq	SINGLE	ILLUMINA	Illumina HiSeq 2000	PRJNA874012	Solanum tuberosum subsp. andigenum	BK_SL1-2
SRR21209922	RNA-Seq	SINGLE	ILLUMINA	Illumina HiSeq 2000	PRJNA874012	Solanum tuberosum subsp. andigenum	BL_SR1-2
SRR21209921	RNA-Seq	SINGLE	ILLUMINA	Illumina HiSeq 2000	PRJNA874012	Solanum tuberosum subsp. andigenum	BM_TL2-2
SRR21209920	RNA-Seq	SINGLE	ILLUMINA	Illumina HiSeq 2000	PRJNA874012	Solanum tuberosum subsp. andigenum	BN_TR2-2
SRR21209918	RNA-Seq	SINGLE	ILLUMINA	Illumina HiSeq 2000	PRJNA874012	Solanum tuberosum subsp. andigenum	BO_SL2-2
SRR21209917	RNA-Seq	SINGLE	ILLUMINA	Illumina HiSeq 2000	PRJNA874012	Solanum tuberosum subsp. andigenum	BP_SR2-2
SRR21209916	RNA-Seq	SINGLE	ILLUMINA	Illumina HiSeq 2000	PRJNA874012	Solanum tuberosum subsp. andigenum	CA_TLC-3
SRR21209915	RNA-Seq	SINGLE	ILLUMINA	Illumina HiSeq 2000	PRJNA874012	Solanum tuberosum subsp. andigenum	CB_TRC-3
SRR21209914	RNA-Seq	SINGLE	ILLUMINA	Illumina HiSeq 2000	PRJNA874012	Solanum tuberosum subsp. andigenum	CC_SLC-3
SRR21209913	RNA-Seq	SINGLE	ILLUMINA	Illumina HiSeq 2000	PRJNA874012	Solanum tuberosum subsp. andigenum	CD_SRC-3
SRR21209912	RNA-Seq	SINGLE	ILLUMINA	Illumina HiSeq 2000	PRJNA874012	Solanum tuberosum subsp. andigenum	CE_TL3-3
SRR21209911	RNA-Seq	SINGLE	ILLUMINA	Illumina HiSeq 2000	PRJNA874012	Solanum tuberosum subsp. andigenum	CF_TR3-3
SRR21209910	RNA-Seq	SINGLE	ILLUMINA	Illumina HiSeq 2000	PRJNA874012	Solanum tuberosum subsp. andigenum	CG_SL3-3
SRR21209909	RNA-Seq	SINGLE	ILLUMINA	Illumina HiSeq 2000	PRJNA874012	Solanum tuberosum subsp. andigenum	CH_SR3-3
SRR21209907	RNA-Seq	SINGLE	ILLUMINA	Illumina HiSeq 2000	PRJNA874012	Solanum tuberosum subsp. andigenum	CI_TL1-3
SRR21209906	RNA-Seq	SINGLE	ILLUMINA	Illumina HiSeq 2000	PRJNA874012	Solanum tuberosum subsp. andigenum	CJ_TR1-3
SRR21209905	RNA-Seq	SINGLE	ILLUMINA	Illumina HiSeq 2000	PRJNA874012	Solanum tuberosum subsp. andigenum	CK_SL1-3
SRR21209904	RNA-Seq	SINGLE	ILLUMINA	Illumina HiSeq 2000	PRJNA874012	Solanum tuberosum subsp. andigenum	CL_SR1-3
SRR21209903	RNA-Seq	SINGLE	ILLUMINA	Illumina HiSeq 2000	PRJNA874012	Solanum tuberosum subsp. andigenum	CM_TL2-3
SRR21209902	RNA-Seq	SINGLE	ILLUMINA	Illumina HiSeq 2000	PRJNA874012	Solanum tuberosum subsp. andigenum	CN_TR2-3
SRR21209901	RNA-Seq	SINGLE	ILLUMINA	Illumina HiSeq 2000	PRJNA874012	Solanum tuberosum subsp. andigenum	CO_SL2-3
SRR21209900	RNA-Seq	SINGLE	ILLUMINA	Illumina HiSeq 2000	PRJNA874012	Solanum tuberosum subsp. andigenum	CP_SR2-3
SRR10153129	RNA-Seq	PAIRED	ILLUMINA	Illumina HiSeq 2000	PRJNA573056	Solanum tuberosum subsp. andigenum	GSM4087781
SRR10153130	RNA-Seq	PAIRED	ILLUMINA	Illumina HiSeq 2000	PRJNA573056	Solanum tuberosum subsp. andigenum	GSM4087782
SRR11920091	RNA-Seq	SINGLE	ILLUMINA	Illumina HiSeq 4000	PRJNA637104	Solanum tuberosum	Kalkaska Control 10 hrs
SRR11920079	RNA-Seq	SINGLE	ILLUMINA	Illumina HiSeq 4000	PRJNA637104	Solanum tuberosum	Kalkaska Control 18 hrs
SRR11920061	RNA-Seq	SINGLE	ILLUMINA	Illumina HiSeq 4000	PRJNA637104	Solanum tuberosum	Kalkaska Control 2 hrs
SRR11920065	RNA-Seq	SINGLE	ILLUMINA	Illumina HiSeq 4000	PRJNA637104	Solanum tuberosum	Kalkaska Control 20 hrs
SRR11920063	RNA-Seq	SINGLE	ILLUMINA	Illumina HiSeq 4000	PRJNA637104	Solanum tuberosum	Kalkaska Control 24 hrs
SRR11920089	RNA-Seq	SINGLE	ILLUMINA	Illumina HiSeq 4000	PRJNA637104	Solanum tuberosum	Kalkaska Control 4 hrs
SRR11920087	RNA-Seq	SINGLE	ILLUMINA	Illumina HiSeq 4000	PRJNA637104	Solanum tuberosum	Kalkaska Control 6 hrs
SRR11920085	RNA-Seq	SINGLE	ILLUMINA	Illumina HiSeq 4000	PRJNA637104	Solanum tuberosum	Kalkaska Control 8 hrs
SRR11920090	RNA-Seq	SINGLE	ILLUMINA	Illumina HiSeq 4000	PRJNA637104	Solanum tuberosum	Kalkaska Thaxomin A Treatment 10 hrs
SRR11920068	RNA-Seq	SINGLE	ILLUMINA	Illumina HiSeq 4000	PRJNA637104	Solanum tuberosum	Kalkaska Thaxomin A Treatment 18 hrs
SRR11920060	RNA-Seq	SINGLE	ILLUMINA	Illumina HiSeq 4000	PRJNA637104	Solanum tuberosum	Kalkaska Thaxomin A Treatment 2 hrs
SRR11920064	RNA-Seq	SINGLE	ILLUMINA	Illumina HiSeq 4000	PRJNA637104	Solanum tuberosum	Kalkaska Thaxomin A Treatment 20 hrs

SRR11920062	RNA-Seq	SINGLE	ILLUMINA	Illumina HiSeq 4000	PRJNA637104	Solanum tuberosum	Kalkaska Thaxomin A Treatment 24 hrs
SRR11920088	RNA-Seq	SINGLE	ILLUMINA	Illumina HiSeq 4000	PRJNA637104	Solanum tuberosum	Kalkaska Thaxomin A Treatment 4 hrs
SRR11920086	RNA-Seq	SINGLE	ILLUMINA	Illumina HiSeq 4000	PRJNA637104	Solanum tuberosum	Kalkaska Thaxomin A Treatment 6 hrs
SRR11920084	RNA-Seq	SINGLE	ILLUMINA	Illumina HiSeq 4000	PRJNA637104	Solanum tuberosum	Kalkaska Thaxomin A Treatment 8 hrs
SRR2126855	RNA-Seq	PAIRED	ILLUMINA	Illumina HiSeq 2500	PRJNA290800	Solanum tuberosum	Solanum tuberosum petiole under long day photoperiod
SRR2126856	RNA-Seq	PAIRED	ILLUMINA	Illumina HiSeq 2500	PRJNA290800	Solanum tuberosum	Solanum tuberosum petiole under long day photoperiod
SRR2126915	RNA-Seq	PAIRED	ILLUMINA	Illumina HiSeq 2500	PRJNA290800	Solanum tuberosum	Solanum tuberosum petiole under long day photoperiod
SRR2126975	RNA-Seq	PAIRED	ILLUMINA	Illumina HiSeq 2500	PRJNA290800	Solanum tuberosum	Solanum tuberosum petiole under long day photoperiod
SRR2127278	RNA-Seq	PAIRED	ILLUMINA	Illumina HiSeq 2500	PRJNA290800	Solanum tuberosum	Solanum tuberosum petiole under short day photoperiod
SRR2127279	RNA-Seq	PAIRED	ILLUMINA	Illumina HiSeq 2500	PRJNA290800	Solanum tuberosum	Solanum tuberosum petiole under short day photoperiod
SRR2127280	RNA-Seq	PAIRED	ILLUMINA	Illumina HiSeq 2500	PRJNA290800	Solanum tuberosum	Solanum tuberosum petiole under short day photoperiod
SRR2127289	RNA-Seq	PAIRED	ILLUMINA	Illumina HiSeq 2500	PRJNA290800	Solanum tuberosum	Solanum tuberosum petiole under short day photoperiod
SRR2131060	RNA-Seq	PAIRED	ILLUMINA	Illumina HiSeq 2500	PRJNA290800	Solanum tuberosum	Solanum tuberosum phloem associated tissue in petiole
SRR2131063	RNA-Seq	PAIRED	ILLUMINA	Illumina Genome Analyzer II	PRJNA290800	Solanum tuberosum	Solanum tuberosum phloem associated tissue in petiole
SRR2131065	RNA-Seq	SINGLE	ILLUMINA	Illumina Genome Analyzer II	PRJNA290800	Solanum tuberosum	Solanum tuberosum phloem associated tissue in petiole
SRR2131064	RNA-Seq	SINGLE	ILLUMINA	Illumina Genome Analyzer II	PRJNA290800	Solanum tuberosum	Solanum tuberosum phloem associated tissue in stem
SRR2131061	RNA-Seq	PAIRED	ILLUMINA	Illumina HiSeq 2500	PRJNA290800	Solanum tuberosum	Solanum tuberosum phloem associated tissue in stem
SRR2131062	RNA-Seq	PAIRED	ILLUMINA	Illumina Genome Analyzer II	PRJNA290800	Solanum tuberosum	Solanum tuberosum phloem associated tissue in stem
SRR11920083	RNA-Seq	SINGLE	ILLUMINA	Illumina HiSeq 4000	PRJNA637104	Solanum tuberosum	Tundra Control 10 hrs
SRR11920081	RNA-Seq	SINGLE	ILLUMINA	Illumina HiSeq 4000	PRJNA637104	Solanum tuberosum	Tundra Control 18 hrs
SRR11920074	RNA-Seq	SINGLE	ILLUMINA	Illumina HiSeq 4000	PRJNA637104	Solanum tuberosum	Tundra Control 2 hrs
SRR11920078	RNA-Seq	SINGLE	ILLUMINA	Illumina HiSeq 4000	PRJNA637104	Solanum tuberosum	Tundra Control 20 hrs
SRR11920076	RNA-Seq	SINGLE	ILLUMINA	Illumina HiSeq 4000	PRJNA637104	Solanum tuberosum	Tundra Control 24 hrs
SRR11920072	RNA-Seq	SINGLE	ILLUMINA	Illumina HiSeq 4000	PRJNA637104	Solanum tuberosum	Tundra Control 4 hrs
SRR11920070	RNA-Seq	SINGLE	ILLUMINA	Illumina HiSeq 4000	PRJNA637104	Solanum tuberosum	Tundra Control 6 hrs
SRR11920067	RNA-Seq	SINGLE	ILLUMINA	Illumina HiSeq 4000	PRJNA637104	Solanum tuberosum	Tundra Control 8 hrs
SRR11920082	RNA-Seq	SINGLE	ILLUMINA	Illumina HiSeq 4000	PRJNA637104	Solanum tuberosum	Tundra Thaxomin A Treatment 10 hrs
SRR11920080	RNA-Seq	SINGLE	ILLUMINA	Illumina HiSeq 4000	PRJNA637104	Solanum tuberosum	Tundra Thaxomin A Treatment 18 hrs
SRR11920073	RNA-Seq	SINGLE	ILLUMINA	Illumina HiSeq 4000	PRJNA637104	Solanum tuberosum	Tundra Thaxomin A Treatment 2 hrs
SRR11920077	RNA-Seq	SINGLE	ILLUMINA	Illumina HiSeq 4000	PRJNA637104	Solanum tuberosum	Tundra Thaxomin A Treatment 20 hrs
SRR11920075	RNA-Seq	SINGLE	ILLUMINA	Illumina HiSeq 4000	PRJNA637104	Solanum tuberosum	Tundra Thaxomin A Treatment 24 hrs
SRR11920071	RNA-Seq	SINGLE	ILLUMINA	Illumina HiSeq 4000	PRJNA637104	Solanum tuberosum	Tundra Thaxomin A Treatment 4 hrs
SRR11920069	RNA-Seq	SINGLE	ILLUMINA	Illumina HiSeq 4000	PRJNA637104	Solanum tuberosum	Tundra Thaxomin A Treatment 6 hrs
SRR11920066	RNA-Seq	SINGLE	ILLUMINA	Illumina HiSeq 4000	PRJNA637104	Solanum tuberosum	Tundra Thaxomin A Treatment 8 hrs

9 Comparison Genome Assembly

Genome Assembly	RH89-039-16	DMv6.1	Solynthus	M6 (v5)	DC(1194)
Sequencing technology	10xG + ONT + CCS + Hi-C	ONT+Hi-C	ONT+Illumina	Illumina	Pacbio HiFi+Hi-C
N50	66.116.962	59.670.755	63.701.590	53.122.106	50.589.760
L50	11	6	6	6	19
Total size	1.673.555.276	741.585.035	716.171.447	676.284.973	2.369.577.969
Largest Contig	97.287.411	88.591.686	72.008.707	85.473.275	98.785.543
Total Contigs	3.024	288	12	533	1.194
% Complete BUSCO embriophyta (n=1614)	99,32	99,38	98,14	97,77	99,38
% Complete and Duplicated BUSCO embriophyta (n=1614)	68,22	1,92	2,85	2,54	87,79
% Complete BUSCO solanales (n=5950)	98,87	98,67	97,13	97,48	98,96
% Complete and Duplicated BUSCO solanales (n=5950)	69,98	2,32	3,33	2,96	89,26
LAI	6,47	13,56	12,86	13,40	13,53

Tab. S4: Comparative Genome Assembly Statistics for Solanum tuberosum Chromosome-Scale Assemblies

10 Functional Annotation

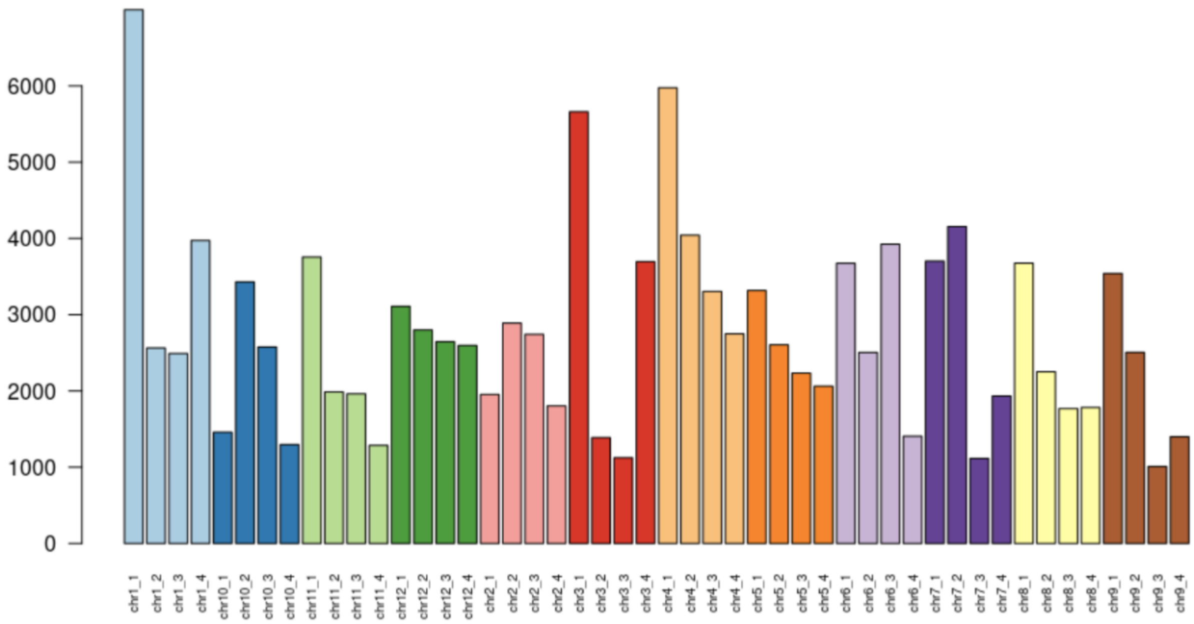

Fig. S22: Barplot illustrating gene predictions distributed across individual chromosomes. Each bar represents the count of gene predictions for a specific chromosome within the genome assembly.

11 Andigenum genomes in pangenome efforts on March 2024

Accession number	Assembly	Provenance of material	Publication
(PI 558142)	No	Argentina	
(PI 258874)	No		Hardigan MA, et al. (2017) Genome diversity of tuber-bearing Solanum uncovers complex evolutionary history and targets of domestication in the cultivated potato. Proceedings of the National Academy of Sciences 114(46):E9999.
(PI 546023)	No	Bolivia	
(PI 245935)	No		
(PI 245940)	No	Chile	
(CIP 700921)	Yes ADG1		Kyriakidou M, Anglin NL, Ellis D, Tai HH, & Strömvik MV (2020) Genome assembly of six polyploid potato genomes. Scientific Data 7(1):88.
(CIP 702853)	Yes ADG2		
(PI 365345)	No		
(PI 607886)	No	Peru	Hardigan MA, et al. (2017)

Tab. S5: Solanum tuberosum group Andigenum (Andigena) genomes (Bozan et al., 2023) and the origin of the material sourced from Genesys (https://www.genesys-pgr.org/). The genome sequencing data for most of these accessions comes from the Illumina platform, except for ADG1 from Peru (blue background).)
